# Extreme specificity of NCR gene expression in *Medicago truncatula*

**DOI:** 10.1186/1471-2164-15-712

**Published:** 2014-08-25

**Authors:** Ibtissem Guefrachi, Marianna Nagymihaly, Catalina I Pislariu, Willem Van de Velde, Pascal Ratet, Mohamed Mars, Michael K Udvardi, Eva Kondorosi, Peter Mergaert, Benoît Alunni

**Affiliations:** Institut des Sciences du Végétal, Centre National de la Recherche Scientifique UPR2355, 91198 Gif-sur-Yvette, France; Research Unit Biodiversity & Valorization of Arid Areas, Bioressources (BVBAA), Faculty of Sciences, Gabès University, Erriadh-Zrig, 6072 Gabès, Tunisia; Institute of Biochemistry, Hungarian Academy of Sciences, Biological Research Centre, 6726 Szeged, Hungary; Samuel Roberts Noble Foundation, Ardmore, Oklahoma 73401 USA; Département de Biologie, Université Paris Sud 11, 91400 Orsay, France; Ablynx, Technologiepark 21, 9052 Zwijnaarde, Belgium

**Keywords:** Symbiosis, Legume nitrogen fixation, Nodulation, Bacteroid, *Medicago truncatula*, *Sinorhizobium meliloti*, NCR, Defensin, Gene expression, Transcriptome compendium

## Abstract

**Background:**

Legumes form root nodules to house nitrogen fixing bacteria of the rhizobium family. The rhizobia are located intracellularly in the symbiotic nodule cells. In the legume *Medicago truncatula* these cells produce high amounts of Nodule-specific Cysteine-Rich (NCR) peptides which induce differentiation of the rhizobia into enlarged, polyploid and non-cultivable bacterial cells. NCRs are similar to innate immunity antimicrobial peptides. The NCR gene family is extremely large in *Medicago* with about 600 genes.

**Results:**

Here we used the *Medicago truncatula* Gene Expression Atlas (MtGEA) and other published microarray data to analyze the expression of 334 NCR genes in 267 different experimental conditions. We find that all but five of these genes are expressed in nodules but in no other plant organ or in response to any other biotic interaction or abiotic stress tested. During symbiosis, none of the genes are induced by Nod factors. The NCR genes are activated in successive waves during nodule organogenesis, correlated with bacterial infection of the nodule cells and with a specific spatial localization of their transcripts from the apical to the proximal nodule zones. However, NCR expression is not associated with nodule senescence. According to their Shannon entropy, a measure expressing tissue specificity of gene expression, the NCR genes are among the most specifically expressed genes in *M. truncatula*. Moreover, when activated in nodules, their expression level is among the highest of all genes.

**Conclusions:**

Together, these data show that the NCR gene expression is subject to an extreme tight regulation and is only activated during nodule organogenesis in the polyploid symbiotic cells.

**Electronic supplementary material:**

The online version of this article (doi:10.1186/1471-2164-15-712) contains supplementary material, which is available to authorized users.

## Background

Legume plants establish a symbiotic relationship with nitrogen fixing soil bacteria, known as rhizobia. For the purpose of this symbiosis, the plant host forms new, specific organs on its roots called nodules, inside which the symbiotic rhizobia are housed, fix nitrogen (i.e. the enzymatic reduction of nitrogen gas to ammonium) and transfer the ammonium to the plant. Nodules contain several thousand endoreduplicated giant symbiotic cells, which are each infected with thousands of intracellular rhizobia. These symbiotic cells are adapted to the symbiosis, to the metabolic exchange with the nitrogen fixing rhizobia and to the intracellular accommodation of this large bacterial population. The symbiotic cells originate from dividing progenitor cells in the nodule meristem. A key step in the differentiation of the symbiotic cells is the exit of the cell division cycle of nodule meristematic cells and a switch to an endoreduplication cycle in these post-meristematic cells. An endoreduplication cycle is a modified cell cycle with repeated replication of the genome without mitosis and cytokinesis, resulting in polyploid cells with increased DNA content and cell volume. The cell cycle switch is under the control of the Anaphase Promoting Complex (APC) and its activator Ccs52A [1, 2]. The differentiating symbiotic cells are gradually infected and filled with rhizobia. Bacteria are released in the host cells through an endocytosis-like process releasing the intracellular bacteria in organelle-like structures called symbiosomes. Mature symbiotic cells have about 80-fold larger cell volume and endoploidy levels up to 64C compared to the diploid (2C) progenitor cells. Their cytosolic space is entirely packed with symbiosomes and their physiology is adapted for symbiosis, feeding the microsymbionts and assimilating and transporting the fixed nitrogen.

Remarkably, Wildermuth [3] noticed, by comparing different biotrophic interactions of plants, that host cell polyploidy is a common feature of symbiotic interactions with rhizobium bacteria and arbuscular mycorrhizal fungi, as well as parasitic interactions with fungi and nematodes. Even in symbiotic interactions of insects with endosymbiotic bacteria, host cells that house the endosymbionts are endoreduplicated cells (e.g. [4]). Thus polyploid host cells may be a well suited adaptation as an interaction site for nutrient exchange with symbiotic microorganisms and some parasites may have evolved to exploit this.

Synchronously with the differentiation of their host cells, the symbiosome bacteria differentiate into nitrogen-fixing bacteria called bacteroids. These bacteroids have a specific physiology and metabolism adapted to the symbiotic life and nitrogen fixation which are dramatically different from those of a free-living bacterium [5]. Interestingly, the differentiation of bacteroids is often accompanied by a morphological and cytological metamorphosis whereby the bacteroid cell becomes enlarged, its envelope fragilized and its genome amplified (polyploid) and condensed [6, 7]. In *Medicago truncatula*, a class of peptides named NCRs (Nodule-specific Cysteine-Rich Peptides) controls the bacteroid elongation and polyploidization [8]. The NCR peptides are produced by the infected symbiotic cells and are transported to the bacteroid-containing symbiosomes. The NCR peptides can induce bacterial elongation and polyploidization *in vitro* on cultured rhizobium or *in planta* when expressed in transgenic *Lotus japonicus* plants which lack NCRs and form non-elongated bacteroids without genome amplification [8]. Some NCRs accumulate to a significant extent in the cytosol of mature bacteroids [8] suggesting that these peptides may have additional functions, other than inducing the morphological transformation and notably, it has been suggested that these intracellular NCRs may affect the bacteroid metabolism [5]. Indeed, it has been demonstrated for the peptide NCR247 that it has multiple bacterial targets leading to inhibition of cell division and affecting the bacterial transcriptome and translation that collectively contribute to the altered physiology of the endosymbionts [9–11].

NCRs are similar to the defensin-type of antimicrobial peptides, and some NCR peptides have antimicrobial activity, killing rhizobium and other bacteria when applied at high concentration [8, 9, 12, 13]. Defensins and other types of antimicrobial peptides are found in all eukaryotes where they are part of the first line of defense against invading microbes. Thus the NCR peptides likely evolved from the ancestral immune repertoire. NCR genes were originally thought to be unique to the IRLC legume clade [14]. The bacteroids in the nodules of the tested species of this clade all share the elongation and polyploidization feature [6]. However, refined bioinformatics tools for the prediction of small peptides in genome sequences led to the discovery of three putative *Arabidopsis* genes that encode peptides with the typical pattern of cysteine residues of the NCRs [15]. The existence of multiple NCR genes in several species of the IRLC clade suggests that the ancestral genes may have gained a new function in symbiosis in the common ancestor of IRLC and that increasing its copy number through gene duplications may have conferred a selective advantage. To counteract the antimicrobial activity of the NCR peptides, *Sinorhizobium meliloti*, the symbiont of *Medicago*, requires the BacA protein. In the absence of this protein, the bacteroids are immediately killed by the NCR peptides as soon as they are released in the symbiosomes in nodule cells [12].

A striking and unusual feature of the NCR gene family in *M. truncatula* is that it is composed of about 600 genes which are seemingly all expressed in the nodules [14–18]. *In situ* expression analysis of individual NCR genes or microarray analysis of a large subset of the family has demonstrated that they are all expressed in the symbiotic nodule cells [8, 14, 18, 19]. Moreover, EST analysis and microarray experiments, testing a number of different plant organs as well as different growth conditions, revealed NCR gene expression only in nodules [14, 18, 19].

The *Medicago truncatula* Gene Expression Atlas (MtGEA) [20, 21] was generated with the whole genome Affymetrix Medicago Gene Chip and compiles microarray data for the majority of *M. truncatula* genes (50,900 probe-sets) over a large set of experiments (254 different experiments in MtGEA version 3). The compendium is a unique and currently the richest resource for analysing gene expression in *M. truncatula*. In this study, we used the MtGEA compendium and additional unpublished and published microarray experiments [22, 23] to describe in great detail the expression profiles of the majority of the *M. truncatula* NCR genes. We show that this gene family has an extreme tissue specific expression profile with undetectable expression in all tissues and conditions except in nodules where they become transcriptionally active to very high levels. In addition, promoter-GUS plants were produced for three NCR genes as well as a specific antibody for one NCR peptide. These tools were used to confirm expression pattern specificity in various conditions, most particularly during biotic interactions.

## Results

### Global analysis of NCR gene expression

**Figure 1:**
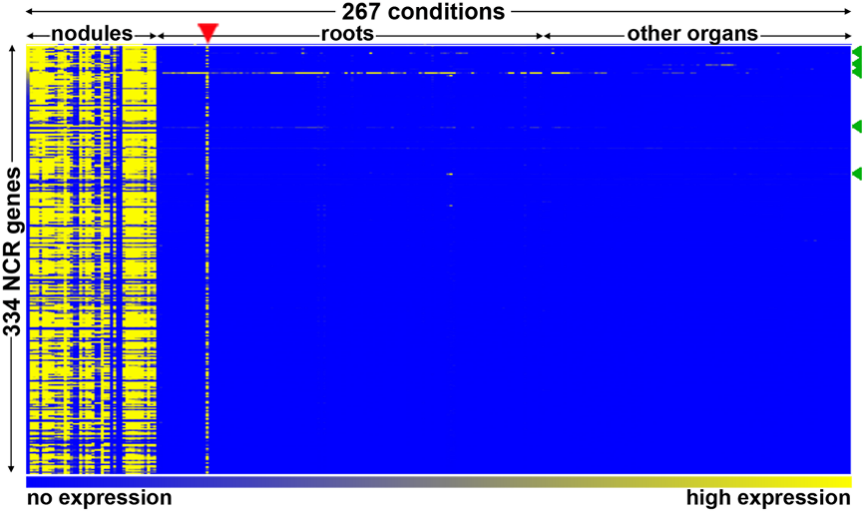
**Heat map of NCR gene expression in the MtGEA compendium.** The heat map shows the expression of 334 NCR genes (rows) in 267 experimental conditions (columns). Experiments are ordered as indicated above the columns. The color scale bar indicates the expression level from background level (blue) to maximum level (yellow). The red arrowhead indicates the mycorrhizal sample that is contaminated with nodules. The green arrowheads locate the NCR genes with relaxed specificity; from top to down: NCR247, NCR235, NCR122, NCR218 and NCR077. The dataset used for the generation of the heat map is provided in Additional file 1: Table S1.

The probe-sets and expression data corresponding to the NCRs described before [14, 16] were searched in MtGEA (version 3) using the BLAST search option of the database. Expression profiles of 334 probe-sets were obtained (Additional file 1: Table S1) and their expression patterns in 267 different experimental conditions (254 from MtGEAv3, five unpublished conditions, three from [22] and five from [23]) are summarised in Figure 1. The transcriptome compendium is mostly derived from the *M. truncatula* genotype ‘Jemalong’ but also contains data sets obtained from the genotypes ‘R108’ and ‘F83005.5’ although all specific experiments discussed below were obtained with the ‘Jemalong’ genotype. As specified in more detail below, the compendium covers the plant’s major organs, various kinds of abiotic and biotic stresses and data from specific cell and tissue types. In the heat map of Figure 1, the experiments are organized in three major groups, namely the profiles of nodule samples, root samples and samples of all other plant organs regardless of the treatment they were submitted to. An obvious global pattern instantly revealed by the heat map is that the nearly complete 334 NCR gene set is only expressed in nodules except for one experiment marked with the red arrowhead, which is a sample annotated in the MtGEA compendium as mycorrhizal roots but is contaminated with nodules (Additional file 2: Figure S1).

The expression profiles of individual NCR genes show expression to very high levels in the nodule conditions and only background levels in the other experiments (Additional file 2: Figure S2A,B). Such profiles are typical for nearly all NCR genes (Additional file 1: Table S1) but by surveying all NCR probe-sets, 5 exceptions were discovered with more or less relaxed nodule specific expression patterns (Figure 1, green arrowheads; Additional file 2: Figure S2C-G). NCR247 and NCR077 are still mainly expressed in nodules but are also weakly active in other conditions. The NCR247 gene seems to be expressed in different root samples and in some samples from aerial tissues although at lower levels than in nodules. It is not evident from the available information of the different experiments to determine what may activate this expression. NCR077 has a higher than usual background level, possibly because of a less specific probe set, but the gene seems to be also expressed in some mycorrhizal samples (Additional file 2: Figure S2D) including a laser-capture microdissection (LCM) sample of arbuscule-containing cells [24]. NCR218 and NCR122 on the other hand have a completely relaxed specificity and are expressed to similar levels in nodules and in other conditions, mostly roots (Additional file 2: Figure S2E,F). NCR235 expression is similarly specific to most other NCR genes except for a weak expression in stems and shoots, which is about 10- to 100-fold lower than in nodules (Additional file 2: Figure S2G). Also NCR247 is expressed in some of the stem samples. Thus, except for these 5 genes, the complete tested NCR gene set is only expressed in nodules and in none of the other conditions that are present in MtGEA.

### Spatio-temporal expression of NCR genes in nodules

**Figure 2:**
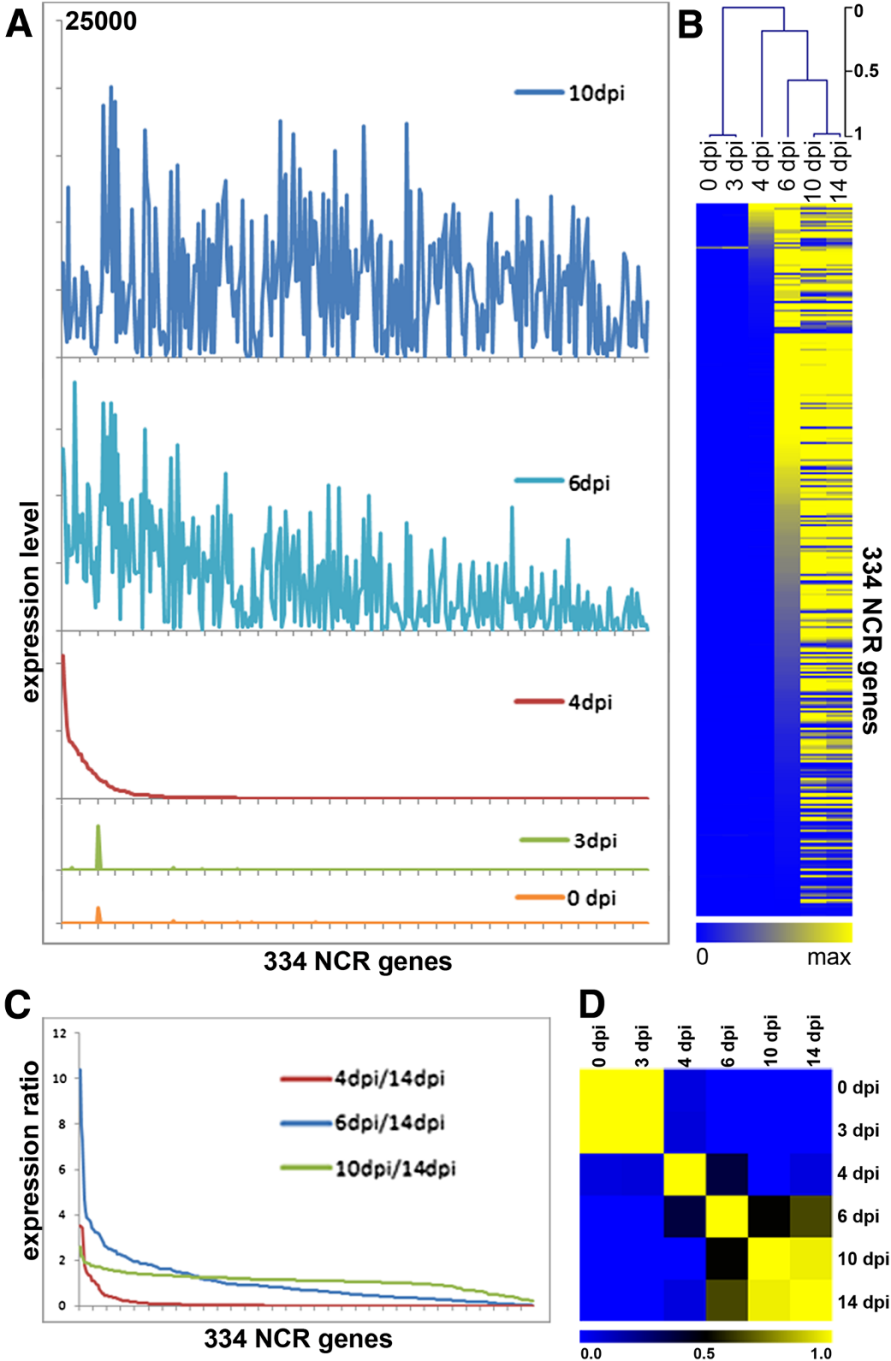
**Successive activation of NCR genes during nodule development. (A)** Expression level of the 334 NCR probe-sets (x-axis) at 0, 3, 4, 6 and 10 dpi. The y-axis scale is the same for all graphs and is 25,000 at maximum. **(B)** Heat map of the same expression data as in (A). **(C)** The ratios of NCR expression levels at 4 dpi, 6 dpi or 10 dpi compared to 14 dpi. For each time point, the genes were ordered from high ratio to low ratio. At 4 dpi, only few NCR genes are activated; at 6 dpi most are activated but not yet at maximum scale; at 10 dpi, nearly all NCR genes are activated at similar level to 14 dpi (ratio close to 1). **(D)** Heat map of the Pearson correlation of the NCR expression profiles at 0, 3, 4, 6, 10 and 14 dpi.

The MtGEA compendium contains 42 different nodule samples including samples of wild type nodules harvested at different days post inoculation with *S. meliloti* and thus at different stages of nodule development. In uninoculated root samples and nodule primordia of 3 dpi, none of the NCR genes are activated (Figure 2A,B). The NCR transcriptomes at 0 dpi and 3 dpi have a correlation coefficient close to 1 (Figure 2D). This suggests that NCRs have no function in the early stages of the interaction. In agreement with this, Nod factor or Myc factor treatments [25] do not induce NCR gene expression (Additional file 2: Figure S3). Nod factors and Myc factors are similar lipochitooligosaccharide signals, produced by rhizobium and mycorrhizal fungi symbionts inducing in the legume host the early stages of nodule and mycorrhizal formation respectively [26, 27]. A subset of about 70 NCR genes is activated at 4 dpi with *S. meliloti* (Figure 2). Some of these genes are already activated at their maximal level while others are only induced to a fraction of their maximal level and reach full expression at a more advanced stage of nodule development (Figure 2). At 6 dpi, most genes are activated but many of them not yet at their maximal level (Figure 2A-C). At 10 dpi the NCR transcriptome seems to be fully activated, to a similar extent as a later time point at 14 dpi. The NCR transcriptomes at 10 dpi and 14 dpi have a correlation coefficient close to 1 (Figure 2D). This pattern hints at a link between the activation of NCR gene expression and the progression of bacterial infection in the incipient nodules.

**Figure 3:**
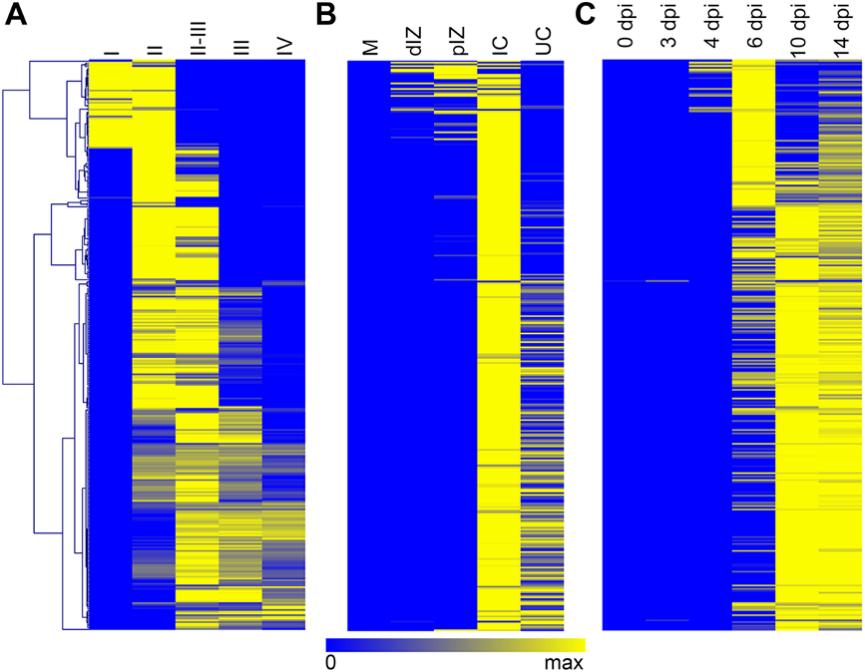
**Spatio-temporal expression of the NCR gene family in nodules. (A)** Heat map and hierarchical clustering of NCRs in function of their spatial expression in nodules (samples I to IV, from the youngest, most apical part of the nodules to the oldest, most proximal part). Clustering was performed using Pearson correlation. **(B)** Heat map of NCR expression profiles obtained by LCM coupled to Affymetrix microarray analysis described by Limpens et al. [23]. M corresponds to meristem, dIZ to the distal zone II or infection zone, pIZ to the proximal zone II, IC to infected cell and UC to uninfected cell. **(C)** Heat map of NCR expression in function of the nodule age (dpi). The genes in the rows in (B) and (C) are in the same order as in panel (A). For each probe-set in the 3 panels, the relative expression was converted to the log2 value of the ratio representing the zone-specific expression over the average expression in all five zones.

Because of the presence of an apical meristem, nodules in *M. truncatula* are of the so-called indeterminate type and all stages of symbiotic cell differentiation, from undifferentiated meristem cells till fully differentiated and functional symbiotic cells, are present in any mature nodule, independent of its age. Therefore, mature nodules are organized in well-defined histological zones: zone I, meristem; zone II, zone of infection and differentiation (polyploidization) of the symbiotic cells; interzone II–III, characterized by amyloplast accumulation; zone III, nitrogen fixation zone with mature functional symbiotic cells; zone IV, senescent zone [7]. This nodule structure suggests that the temporal NCR expression profiles defined above could correlate with a spatial pattern in the nodule tissues. To test this possibility, we analyzed transcriptome data from 4-weeks-old nodules that were hand-sectioned in five different parts (see Methods section). These samples correspond to the nodule tissues from the most apical part of the nodule to the most proximal part and are approximately overlapping with the nodule zones I, II, II-III, III and IV. Cluster analysis of the NCR abundance profiles in these five samples distinguishes groups of NCR genes that have preferential expression in defined zones of the nodule and that are sequentially activated from nodule apex to proximal tissues (Figure 3A and Additional file 2: Figure S4). The transcriptome in specific tissues and cells of nodules was also obtained by LCM [23]. In this experiment, the transcriptome was analyzed in nodule meristems, the distal and the proximal infection zone, the infected cells of the fixation zone and the uninfected cells of the fixation zone (Figure 3B). Although the type of samples are not entirely overlapping, a good correspondence can be observed between the LCM dataset and the hand-dissected dataset (Figure 3A,B). The LCM dataset shows that NCR genes are not expressed in the nodule meristem which is free of rhizobia (Figure 3B). This observation is in agreement with their activation by the infecting rhizobia. It also indicates that the expression of the early NCR genes in the hand-dissected sample I results from the significant presence of cells from the infection zone in that sample. NCR genes were reported to be expressed in infected cells [7]. Unexpectedly, the LCM dataset revealed a relatively high expression of several NCR genes in the uninfected cells (Figure 3B, sample UC). However, among them were the genes NCR001, NCR084, NCR035 and NCR247 for which *in situ* hybridization, promoter-GUS or -mCherry fusions or immunolocalization of the peptide demonstrated a specific expression in the infected symbiotic nodule cells and no expression in uninfected cells [8, 10, 14]. Possibly, the signal in the LCM sample of the uninfected cells is the result of some contamination with infected cells or is due to background hybridization coming from the RNA amplification procedure used with the very low amount of RNA obtained from the LCM samples [23]. Nevertheless, when comparing the expression level of all NCRs in the uninfected and infected cells, only two genes had a significant higher expression in the uninfected cells. Strikingly, those two genes are NCR218 (9,6 fold higher; *t*-test P = 0,002) and NCR122 (3,2 fold higher; *t*-test P = 0,018) which are the only 2 NCRs that are consistently expressed to high levels in roots (Additional file 2: Figure S2E,F).

When matching the spatial patterns with the corresponding temporal patterns, a fairly good correspondence can be observed: genes expressed in the apex are mostly also fully activated early during the nodule development at 4 or 6 dpi, while genes expressed in the more proximal tissues are activated late in nodule development (Figure 3). The correspondence between the spatial and temporal pattern of NCR gene expression is also obvious when considering the clusters of genes representing the major expression profiles (Additional file 2: Figure S4). For example, genes of clusters 1 and 2 (Additional file 2: Figure S4) are expressed in the most apical part of the nodule and this correlates with an early activation at 4 or 6 dpi in the temporal pattern. On the other hand, genes in cluster 5 have maximum expression only in sample II-III and this corresponds with an activation late in nodule development at 10 dpi (Additional file 2: Figure S4).

Together these spatio-temporal patterns reveal that the NCR genes are activated in different waves, in agreement with our previous results that identified two key points in nodule development associated with major transcriptional activation, one at the formation of symbiotic cells and another one when bacteroids differentiate [19]. Nevertheless, the present analysis is refining this description and shows that NCR genes are activated in at least 3 waves and moreover they can be distinguished by the maintenance or the decline of their expression in the older nodule cells.

### NCR genes are not directly involved in nodule senescence

**Figure 4:**
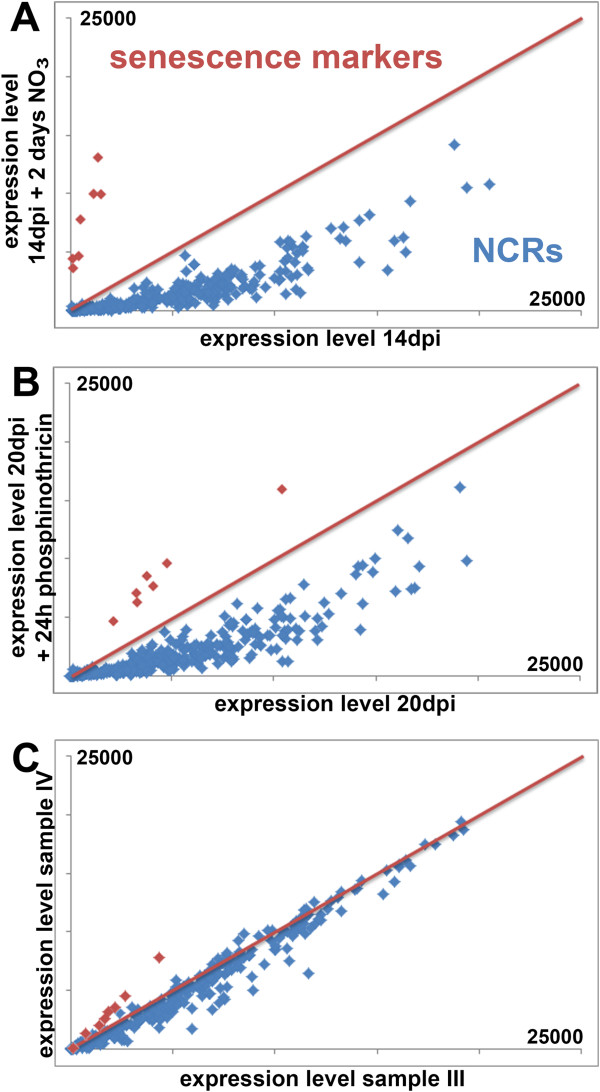
**NCR expression during nodule senescence. (A)** Scatter plot of the expression of 334 NCRs (blue) and 8 senescence marker genes (red) in 14 dpi nodules (x-axis) and 16 dpi nodules, which have been treated before harvest with nodule senescence-inducing nitrate for 2 days (y-axis) [20]. **(B)** Scatter plot of the expression of 334 NCRs (blue) and 8 senescence marker genes (red) in 20 dpi nodules (x-axis) and 20 dpi nodules, which were treated for 24 h with phosphinothricin before harvest (y-axis) [29]. **(C)** Scatter plot of the expression of 334 NCRs (blue) and 8 senescence marker genes (red) in hand-sectioned nodule sample III (x-axis) and in hand-sectioned nodule sample IV (y-axis). The scale of all axes is the same and is 25,000 at maximum. The red line in the three graphs indicates a ratio of 1 between the two conditions that are compared. The probe-sets for the senescence marker genes, encoding cysteine proteinases, a chitinase, a nuclease, a nucleoside transporter and a metal-nicotinamide transporter, are provided in Additional file 1: Table S1.

Because of their resemblance to the defensin-type of antimicrobial peptides [14] and because they have *in vitro* and *in vivo* antimicrobial activity [8, 9, 12, 13], part of the NCR family could be involved in the killing of the rhizobia during the senescence process of nodules as has been suggested before [18]. Nodule senescence leads to a complete digestion of the bacteroids and the symbiotic host cells and is controlled by the transcriptional activation of a battery of genes which are involved the digestion of macromolecules and the remobilization of the liberated nutrients [28]. To test whether NCR genes are induced by senescence, we analyzed their expression in nitrate or herbicide induced senescent nodules [20, 29] (Figure 4; Additional file 2: Figure S5). Besides the NCR genes, 8 senescence marker genes were included in the analysis (Additional file 1: Table S1) [28]. Both treatments induced senescence as indicated by the strong activation of the senescence marker genes. However, all the NCR genes without exception are reduced in expression by the senescence-inducing treatments, in agreement with a recent report [30]. The downregulation of NCR gene expression is induced very rapidly, within 4 hours of nitrate application [30] or 8 hours of phosphinothricin treatment (Additional file 2: Figure S5) and occurs before the degeneration of the symbiotic cells and their bacterial symbionts [29]. The down-regulation of the NCRs most likely reflects the shutdown of the symbiotic process. This expression pattern suggests that none of the NCR genes has a direct role in senescence. This conclusion is also confirmed by comparing the hand-dissected nodule samples III and IV which are enriched for the nitrogen fixation zone III and the senescence zone IV respectively. None of the NCRs is significantly (*t*-test P < 0,05) higher expressed in sample IV (Figure 4C).

### Promoter-GUS analysis and immunolocalization of selected NCRs in nodules

In order to confirm the expression data from MtGEA, stable transgenic *M. truncatula* R108 lines were generated carrying promoter-GUS fusion constructs for 3 different NCR genes, representing different temporal classes of NCRs and inoculated with *Sinorhizobium meliloti* strain 1021 or *Sinorhizobium arboris* strain B554. NCR001 is not activated before the late stages of the nodule formation, NCR084 is slightly induced in early time points (4 dpi) and fully activated at the mature stage of the nodule and finally NCR121 is an early gene which is already fully activated at 4 dpi. GUS expression in the 3 transgenic lines was not detected in root tips or other root parts (Additional file 2: Figure S6). In agreement with its temporal regulation during nodulation, NCR121 expression was induced in young nodule primordia as early as 5dpi and remained expressed throughout the experiment in the entire infection zone and the fixation zone of mature nodules (Additional file 2: Figure S6). NCR084 expression was detected from 11 dpi on and was mainly confined to the proximal infection zone, the interzone II-III and to the distal part of the fixation zone (Additional file 2: Figure S6). NCR001 expression was detectable from 11 dpi in the developing fixation zone and its expression extends in the following days as the fixation zone is growing (Additional file 2: Figure S6). All 3 genes are only expressed in the symbiotic nodule cells. In older nodules, at 30 dpi, displaying a senescence zone, NCR expression was never detected in the senescing tissues, nor was their expression enhanced in the proximal fixation zone adjacent to the senescent tissue (Additional file 2: Figure S6), confirming that NCR genes are not involved in the senescence process. Overall, the temporal and spatial promoter-GUS expression patterns are in very good agreement with the expression profiles deduced from the transcriptome compendium.

The particular expression pattern of NCR122 with its relaxed tissue specificity (Additional file 2: Figure S2F) and its apparent expression in the uninfected nodule cells, together with the availability of an anti-NCR122 antibody prompted us to specifically analyze the localization of the NCR122 peptide in nodules. Immunolocalization of the peptide revealed indeed a specific presence of NCR122 in the uninfected cells of the central zone of a mature nodule as well as in the uninfected cortical cells of the nodule (Additional file 2: Figure S7). Together with the transcriptome data, this indicates that NCR122 and most likely also NCR218 are the only NCR peptides that are specific to uninfected root and nodule cells.

### Expression of NCR genes in plant organs

Previously, NCR expression in conditions other than the symbiosis with rhizobium was tested by EST analysis [14] and with dedicated microarrays [31], indicating the absence of expression. The MtGEA database offers the possibility to extend this analysis to more plant organs and biotic and abiotic stress conditions.

Besides nodules, 8 other plant organs [20] were interrogated for NCR expression (Additional file 2: Figure S8) as well as plant treatments with the phytohormones auxin, cytokinin and auxin transport inhibitors [32, 33] (Additional file 2: Figure S9). Interestingly, treatment of roots with the auxin transport inhibitors TIBA or NPA leads to the formation of uninfected nodule-like structures [33]. However, in none of these conditions were NCR genes expressed except for the NCR genes with relaxed expression described above.

NCRs resemble the defensin-type of antimicrobial peptides and plant defensins are often expressed to high levels in “infection-sensitive” organs like flowers or seeds. Because of the complete lack of detectable expression of NCRs in these organs (Additional file 2: Figure S8), they most probably do not have a defensive function in these organs. Nevertheless, many non-NCR defensin-like genes were found to be expressed in seeds, potentially involved in their protection [31].

### Expression of NCR genes after biotic and abiotic stress

Defensins are also induced during infection with pathogens or during salt and drought stresses [34–37]. Therefore, we specifically analyzed how the NCR gene family is expressed during such conditions (Additional file 2: Figure S10 and Additional file 2: Figure S11) [22, 38–45]. Not considering the 5 NCR genes with relaxed specificity, we could not detect expression of any of the NCR genes in all these data sets together (with the possible exception of giant cells formed by the nematode *Meloidogyne incognita*; Additional file 2: Figure S10B). Several NCRs showed a hybridization signal in giant cells although the level was about 1 to 2 orders of magnitude lower than the signal in nodules for the same NCR gene. However, it should be noted that the giant cells were isolated by LCM and that the array hybridization was performed with an amplified cDNA sample [42] which could be a source of background hybridization. In any case, besides the possible exception of the giant cells, the data indicate that the NCR genes seem not to be used by the plant to control infections other than the rhizobium bacteria in nodules.

### Promoter-GUS analysis of NCR expression during pathogenic interactions

We used the 3 NCR promoter-GUS reporter lines to confirm the absence of NCR expression during pathogenic responses and to complement these observations. The MtGEA dataset includes *M. truncatula* responses to root pathogens and therefore we analyzed leaf or stem pathogens that encompass also other trophic interactions and infection strategies. Inoculation of *M. truncatula* leaflets with the necrotrophic soft rotting bacterium *Dickeya dadantii* 3937 induced maceration symptoms from 1 dpi on, but failed to induce NCR expression (Additional file 2: Figure S12). Similarly infiltration of the virulent strain *Pseudomonas syringae* pv. *tomato* DC3000 (*Pst*) induced necrosis in the infiltrated zone within 2 dpi, whereas the *hrcC* mutant strain that is unable to form a functional type three secretion system (TTSS) did not induce any visible reaction (Additional file 2: Figure S12). NCR expression was not detected in the infiltrated leaflets in either condition (Additional file 2: Figure S12). Although *Pst* DC3000 is not described as a natural pathogen of *M. truncatula*, the necrosis induced by the wild type strain and its absence in the presence of the TTSS mutant suggest that at least some bacterial effector proteins can be specifically recognized by the plant resulting in a necrotic response similar to the hypersensitive response it provokes on non-host *Nicotiana benthamiana* plants [46]. Similarly, inoculation of the same *M. truncatula* lines with the necrotrophic polyphagous grey mold-causing fungus *Botrytis cinerea* yielded typical symptoms at 7 dpi without inducing any detectable NCR expression (Additional file 2: Figure S12). The results of our pathoassays are also in line with a recent study showing that NCR expression was not detected during the compatible interaction of *M. truncatula* with the hemibiotrophic leaf pathogen *Colletotrichum trifolii* or with the biotrophic soil pathogen *Phytophtora medicaginis* [31]. Altogether, our data and the study from [31] are in agreement with the MtGEA dataset and broaden the conclusion that NCRs are not involved in pathogen responses, whatever the trophic (bio-, hemibio- or necrotrophic) interaction, the host or non-host status and the output of the interaction (disease or resistance). Finally, as herbivory and more generally wounding may induce plant defenses around the wounded zone, we also tested the effect of mechanical wounding on NCR expression but again no NCR expression could be detected in the wounded leaflet (Additional file 2: Figure S12).

### NCR genes have very high tissue specificity as measured by Shannon entropy

The above analyses reveal an extreme specificity in expression for the NCR gene family: the genes are only expressed in nodules and not in any other organ or physiological condition. To express this specificity quantitatively and to compare it to other types of specifically expressed genes, the complete MtGEA probe-set was analyzed and their Shannon entropy was calculated. Shannon entropy is a metric for characterizing the uniformity of the expression pattern of a gene over the tested conditions [47]. Low entropy values indicate high tissue specificity while high entropy levels characterize ubiquitous expression.

**Figure 5:**
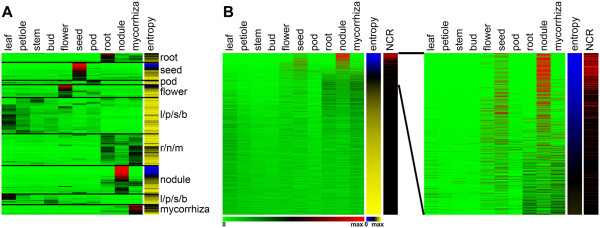
**Shannon entropy. (A)** Hierarchical clustering of the 9,000 probe-sets with the highest tissue specificity (lowest entropy values *E*_*g*_). The expression heat map is in green-black-red colour scheme. The entropy heat map is in blue-black-yellow scheme. The experimental conditions (plant tissues) are in columns and their identities are marked above them. The genes are in rows and the preferential expression of clusters of genes is indicated on their right: ‘l/p/s/b’ indicates two clusters with preferential expression in the aerial tissues leaf, petiole, stem and bud; ‘r/n/m’ indicates a cluster with preferential expression in the underground tissues root, nodule and mycorrhiza. The other clusters labelled ‘root’, ‘seed’, ‘pod’, ‘flower’, ‘nodule’ or ‘mycorrhiza’ correspond to genes with specific expression in one of these tissues only. **(B)** The data set of panel (A) was ordered according to increasing entropy of the genes. The left panel shows the relative expression level of the 9,000 genes in the 10 tissues (green-black-red heat map) and the entropy values *E*_*g*_ (blue-black-yellow heat map). The location of the NCR genes is indicated with the black-red heat map (NCR): red means NCR, black means other gene type. The right panel is an enlargement for the first 1,800 genes (entropy values *E*_*g*_ from 0.07 to 1.64). The scale bar for the expression level heat map is green: 0, black: 0.5 and red: 1 and for the entropy heat map blue: 0, black: 1.44 and yellow: 2.88 (maximum entropy in the complete data set is 3.32 (log_2_(*N*)).

Ten different tissues were taken into consideration: leaf, petiole, stem, bud, flower, seed, pod, root, nodule and mycorrhiza. For nodule, seed and root the mean value of different developmental stages or experiments was used (for “nodule”: 4, 10, 14 and 28 dpi stages; for “seed”: 10, 12, 16, 20, 24 and 36 dap; for “root” the 0 dpi control for nodulation and an independent experiment); thus in total 19 experiments were used. The Shannon entropy was calculated as described (see Methods section) [47, 48] for each of the 50,900 probe-sets using these 10 tissue datasets. The 9000 probe-sets with the lowest entropy (and therefore the highest tissue specificity) were selected for further analysis (Additional file 3: Table S2). A hierarchical cluster analysis of these 9000 genes was calculated (Figure 5A). Clusters of tissue specific genes can be distinguished for root, seed, pod, flower, aerial tissues (l/p/s/b), root tissues (r/n/m), nodule and mycorrhiza. The seed, flower, mycorrhiza and especially the nodule clusters are enriched in genes with low entropy value *E*_*g*_ (Figure 5A). The strong enrichment of nodule-specific genes among the low entropy genes becomes more obvious when re-ordering the dataset according the increasing entropy levels (Figure 5B). This analysis shows that in *M. truncatula* the genes with the lowest entropy and thus the highest tissue specific expression are mostly nodule-specific genes and to a lesser extent, seed- and flower-specific genes. Strikingly, among the nodule-specific genes, the NCRs are the most represented ones (Figure 5B).

Although the NCR family is by far the most represented among the nodule-specific low entropy genes, many other known nodule specific genes have very low entropy (Additional file 3: Table S2). These include for example leghemoglobin genes, the Glycine-Rich Peptide (GRPs) genes [16, 49], the Small Nodulin Acidic RNA-binding Protein (SNARP) gene family [50], genes encoding a small family of secretory calmodulin-like proteins [14, 51], the *DNF2* gene involved in suppression of defense responses in the symbiotic cells [52] and others. Most interestingly, also putative retrotransposons (probe-sets Mtr.9294.1.S1_at and Mtr.636.1.S1_at) and a Dicer 1-like ribonuclease III gene (probe-set Mtr.41531.1.S1_at) are among the nodule specific low entropy genes (Additional file 3: Table S2).

**Figure 6:**
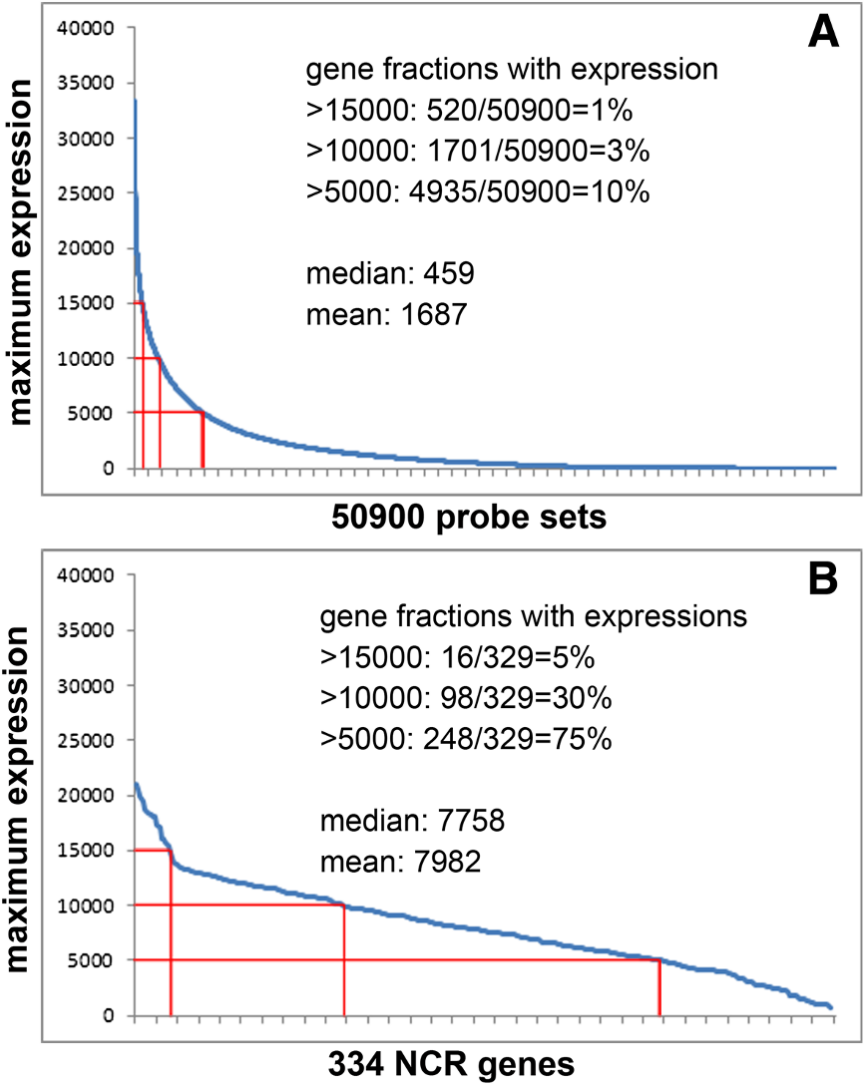
**NCR genes are among the most actively expressed genes in** ***M. truncatula*** **. (A)** Maximum expression level of the 50,900 *M. truncatula* probe-sets. **(B)** Maximum expression level of 334 NCR genes.

Besides the high tissue-specificity, another aspect of expression in which the NCRs stand out from the average *M. truncatula* genes is the strength of expression. We used the hybridization signal on the microarrays as a proxy to strength of gene expression. For each of the 50,900 probe-sets in MtGEA, we searched for its maximal signal in the 267 experiments. These hybridization signals vary from 33,500 for the strongest expressed gene to 9 (background) for the weakest. One percent of the probe-sets have an expression level higher than 15,000, 3% higher than 10,000 and 10% higher than 5,000. The mean signal is 1,687 and the median 459 (Figure 6A). The same analysis on the subset of probes corresponding to the NCR genes gives a completely different picture: 5% of NCR genes have signals above 15,000, 30% above 10,000 and 75% above 5,000 with a mean signal of 7,982 and a median of 7,758 (Figure 6B). Thus, the NCR genes are among both the most specific and the most strongly expressed genes in the genome of *M. truncatula*.

## Discussion

The expression of NCR genes has been studied - in *M. truncatula* mostly but also in some other IRLC legumes - at the level of individual genes by RT-PCR, *in situ* hybridization, immuno-localization and promoter-marker gene fusions or at the family level by EST-analysis, macroarrays, dedicated microarrays or whole-genome microarrays [8, 10, 14, 16, 18, 19, 31, 53]. These studies detected NCR expression only in nodules and in no other tested tissues. By mining publicly available whole-genome transcriptome data, we have extended this analysis of NCR gene expression to a very large number of conditions, together covering most plant organs as well as different growth conditions including biotic and abiotic stresses. As a whole, our study suggests that, apart from 5 genes, all NCRs are only expressed in nodules. Moreover, quantifying the specificity of expression with the Shannon entropy factor reveals that the NCR genes, and more generally, nodule specific genes are among the most specifically expressed genes in *M. truncatula*. This suggests thus that nodulation in *Medicago* is in large part depending on genes solely dedicated to this symbiotic process. These genes may be resulting from gene duplications followed by neo-functionalization (for example the DNF2 protein or the leghemoglobin proteins which have non-symbiotic homologues) or they may be unique for the symbiosis (possibly the NCRs, GRPs, SNARPs and others). In addition to that, the expression of the NCR genes in nodules reaches very high levels. Even if certain NCR genes are expressed at a low level, the majority of them are among the highest expressed genes in the whole genome of *Medicago*. This is in agreement with our previous estimation, based on EST counts, that all NCR mRNAs together constitute almost 5% of the total mRNA population in nodules [14].

In accordance with their resemblance to antimicrobial peptides of the innate immunity such as defensins, many NCR peptides, in particular the most cationic ones, have a strong antimicrobial activity against a diversity of bacteria as well as fungi [8–10; unpublished data]. Despite this, the NCR genes are not expressed in any of the pathogenic interactions of *Medicago* tested here or by [31]. This included interactions with bacteria, fungi, oomycetes and nematodes. They are also not expressed in organs like leaves, seeds and flowers which often express high levels of innate immunity antimicrobial peptides [35]. Therefore, it seems that the NCR peptides have no function in innate immunity.

*In situ* detection of NCR expression has demonstrated for all the tested genes that they are specifically expressed in the symbiotic nodule cells but different subsets of NCR genes are activated at different stages of differentiation of these host cells ([8, 10, 14]; this work). Transcriptome analysis extended this pattern to the whole family. The NCR genes are not activated by Nod factors or during the very early stages of the nodule organogenesis when infected cells are not yet formed (this work; [18, 19]). During the development of wild type nodules, they are activated in consecutive waves and their first appearance coincides with the formation of infected symbiotic cells [19]. We show here that NCR genes are activated during nodule development in at least 3 temporal waves corresponding to specific spatial expression patterns. Genes activated early in nodule development are expressed in the more distal nodule parts (close to the apex) while genes activated late during development are expressed in the proximal nodule tissues. In addition, certain clusters of genes, once activated, maintain their activity when the tissues grow older while other clusters are characterized by a decline of their expression in the older nodule cells. Our spatial analysis of NCR expression is in strong agreement with a recently published study [54] that used LCM of nodule zones coupled to RNA-Sequencing (Additional file 2: Figure S13).

Transcriptome analysis of non-functional nodules that are formed by bacterial or plant symbiotic mutants and that are arrested at different stages of nodule development, is also in agreement with specific expression of all NCR genes in the symbiotic nodule cells: their transcriptional activation is only observed when polyploid symbiotic cells are formed in the mutant nodules [19]. For example, in nodules of the *M. truncatula* TE7 mutant which is affected in the *IPD3* gene [55, 56] and in nodules infected by the *S. meliloti exoY* mutant, no infected and polyploid symbiotic cells are formed and these nodules do not express any of the NCR genes [19]. Conversely, in nodules infected by the *S. meliloti bacA* mutant which contain symbiotic cells with undifferentiated bacteroids, only a subset of NCR genes is activated while in other mutants, forming normal symbiotic cells with differentiated bacteroids, NCR genes are activated to a similar extend as in the wild type [18, 19]. Together, the expression pattern of all the tested NCR genes suggests that the endosymbiotic rhizobia in the symbiotic nodule cells are the only targets of the peptides. However, the distinct spatio-temporal profiles clearly suggest that NCR peptides have many different roles. Subsets of NCR genes that are expressed during the early stages of symbiotic cell formation might be involved in the elongation and polyploidization of the bacteroids while other subsets that are active in later stages of symbiotic cell formation or even after the completion of the symbiotic cell differentiation might have other functions in the bacteroids. Moreover, we find that the expression of the NCRs has been shut down when nodule senescence is activated, meaning that the antimicrobial NCR peptides have no direct role in the lysis and digestion of the bacteroids that is taking place during senescence of nodules.

Very little is known about how the very specific regulation of NCRs is achieved. Since their expression is correlated with bacterial infection of the symbiotic cells, the perception of bacterial signals such as components of the bacterial envelope could be involved. The transcription factor EFD, belonging to the ethylene response factor family, may control, directly or indirectly, the expression of a subset of NCR genes since a mutant forms nodules in which part of the NCR genes are downregulated and in which bacteroid differentiation is partially impaired [57]. The IPD3 protein is another transcription factor that might be involved, directly or more likely indirectly, in the regulation of the NCR genes and the other symbiotic cell specific genes [58, 59]. Indeed, in the *M. truncatula ipd3* mutant nodules, the symbiotic cells do not form and the symbiotic cell specific genes, including the NCRs, are not activated [19]. In agreement with this, the IPD3 gene is expressed in the whole nodule and its expression domain overlaps with the NCR expression zone [60].

Searching for potential *cis*-elements in the promoters of NCR genes with different algorithms yielded 5 different conserved motifs of 41 to 50 bp, which are specifically enriched in the 1000 bp promoter regions [18]. Some of these motifs show resemblance to previously described motifs conferring nodule-specific gene expression. However, the role, if any, of these motifs in the remarkable expression pattern of the NCR genes needs further investigation. Interestingly, some of these motifs comprise Auxin Response Factor binding sites that may suggest a role for auxin in NCR regulation [18]. However, the MtGEA dataset from auxin treated seedlings do not show any NCR induction, pointing out a more complex regulatory mechanism controlling NCR expression.

The very tight regulation of the NCR genes that was revealed here might indicate that besides *cis*- and *trans*-acting factors, regulation at the level of chromatin might also be involved in the activation of the NCR genes. Moreover, endoreduplication seems to be a prerequisite for their activation [19] and might thus, by a presently unknown mechanism, be implicated in the activation of this gene family. In that respect, it is interesting to note that the giant feeding cells induced by the nematode *M. incognita* are highly polyploid cells and possibly express faintly a few NCR genes. Nevertheless, this observation could be an experimental artefact and will require further experimental confirmation.

Genes with high tissue-specific expression are often actively silenced during most of the plant growth by epigenetic mechanisms. Since in *M. truncatula* the nodule-specific genes display the highest level of expression specificity, it might be worthwhile to investigate if epigenetic control is important in the regulation of the symbiotic cell-specific genes. The nodule-specific expression of putative retrotransposons, which are usually epigenetically silenced, and the Dicer 1-like ribonuclease III gene, which may have a role in epigenetic regulation, as well as the identification of small RNAs potentially targeting NCR genes [61] are all in agreement with such an epigenetic control of the symbiotic cell-specific genes.

Plant genomes contain large numbers, several hundreds to thousands, of resistance genes (*R*) of the NB-LRR family that recognize specific pathogen effectors and trigger resistance. Silencing by microRNAs has been proposed as a mechanism to avoid unregulated expression of *R* genes which may be a threat to the plant and represent a fitness cost [62]. Why *M. truncatula* maintains such a large repertoire of NCR genes is not known. It is also not known whether closely related legumes of the IRLC have an arsenal of NCRs of similar size. However, it seems likely to us that expressing such a large gene family might be a fitness cost for the plant that is not to be neglected. Therefore, keeping the whole gene family under a very tight regulatory control might be essential for the plant.

## Conclusions

From the transcriptome data mining and experimental confirmation described here, we can conclude that apart from very few exceptions, the hundreds of NCR genes encoding defensin-like peptides are only activated during nodule formation. They are not expressed in other plant organs, during pathogen attack or abiotic stress. In nodules, they are not yet activated during the very early stages before symbiotic nodule cells are formed and rhizobia are released in symbiosomes within the host cells. NCR genes are also not involved in symbiosome and bacteroid degradation during nodule senescence since their gene expression shuts down when senescence is initiated. However, the expression pattern of NCRs in successive waves during nodule formation suggest that the bacteroids are the only targets of the peptides and that subsets of the peptides might be involved in bacteroid differentiation and other subsets in bacteroid functioning. The NCR genes are among the most specifically expressed genes in *M. truncatula*. Moreover, when activated in nodules, their expression level is among the highest of all genes. Together, these data show that the NCR gene expression is subject to an extreme tight regulation and is only activated during nodule organogenesis in the symbiotic cells.

## Methods

### Analysis of MtGEA data

The MtGEA transcriptome compendium was downloaded from the website of the Samuel Roberts Noble foundation (http://mtgea.noble.org/v3/). The data from [22, 23] were obtained from the NCBI Gene Expression Omnibus (accession n° GSE53406 and GSE43354 respectively). All the data were imported in Excel (Additional file 1: Table S1) for extracting the expression profiles of the 334 NCR probe-sets and for further treatments. The NCR probe-sets on the Affymetrix Medicago GeneChip, which was used for the MtGEA transcriptome compendium, were obtained by BLASTn searches on the MtGEA website (Additional file 1: Table S1). Each individual NCR nucleotide sequence resulted in the identification of multiple probe-sets due to the homology between NCR gene sequences. In total 334 different probe-sets were retrieved. This collection represent likely nearly all NCR probe-sets present on the Affymetrix Medicago GeneChip and the remaining genes identified in Young et al. [17] and Zhou et al. [15] are missing from these arrays because they were not yet annotated at the time of array design.

Cluster analysis of the complete MtGEA dataset was performed using the MeV software package (http://sourceforge.net/projects/mev-tm4/). Briefly, the Excel datasheet extracted from MtGEA was analyzed using the Euclidean distance application with average linkage settings. Heat maps were generated with MeV and histograms and graphs with Excel.

### Entropy calculations

Calculations were performed on the MtGEA dataset in Excel. For the normalization of expression levels in *N* tissues, the relative expression *P*_*t/g*_ of a gene *g* in a tissue *t* was calculated as *P*_*t/g*_ = *W*_*t/g*_/Σ_1≤*t*≤*N*_*W*_*t/g*_ where *W*_*t/g*_ is the expression level of the gene *g* in the tissue *t*. The Shannon entropy *E*_*g*_ of gene *g* is calculated as *E*_*g*_ = Σ_1≤*t*≤N_-*P*_t*/g*_log_2_(*P*_t*/g*_). *E*_*g*_ ranges from zero for genes expressed in a single tissue to log_2_(*N*) for genes expressed uniformly in all tissues considered. Heat maps of entropy values were generated by the MeV software package.

### Transcriptome analysis of hand-dissected nodule zones

Using the leghemoglobin colour gradient along the nodule as guideline, five regions from 28 dpi nodules were hand-dissected as previously described [63]. These samples correspond to the nodule tissues from the most apical part of the nodule with the youngest symbiotic cells to the most proximal part containing the oldest symbiotic cells. Sample I is the meristem and the underlying few cell layers of post-meristematic cells which start the infection and differentiation process. Sample II corresponds mainly to the infection and differentiation zone II. Sample II-III corresponds essentially to the interzone II-III. Sample III is the nitrogen fixation zone III, easily characterized by its pink color due to the accumulation of high amounts of leghemoglobin and finally sample IV is the senescence zone IV that is recognized by its green color resulting from the accumulation of biliverdin, a product of the catabolism of leghemoglobin-derived heme. It should be noted that each of these hand-dissected samples is enriched for the indicated zone but can contain cell layers form the adjacent zones as well.

Total RNA extraction and purification were conducted as described [20]. For hybridization onto the Affymetrix *M. truncatula* Genechip Array probes were synthesized and labelled from 500 ng RNA using the Gene Chip 3′IVT express kit following manufacturer’s guidelines (Affymetrix). Global normalization of expression was carried out using the Robust Multiarray Average Express software [64].

### Transgenic plants and GUS analysis

The promoters of NCR001, NCR084 and NCR121 (respectively 2.5 kb, 1.5 kb and 1 kb fragments upstream of the ATG) were obtained by an Amplified Fragment-Length Polymorphism (AFLP) based PCR protocol as described [65] and recombined in the Gateway vector pDONRP4-P1R according to the manufacturer’s instructions (Invitrogen). Primers used for the amplification and cloning of the promoters were: attB4FWD_NCR001, GGGGACAACTTTGTATAGAAAAGTTGGTTGTCCTTATTAGAGCGCC; attB1REV_NCR001, GGGGACTGCTTTTTTGTACAAACTTGTATGTTTCATCCTTTGAACG; attB4FWD_NCR084, GGGGACAACTTTGTATAGAAAAGTTGGCGAGAAAGGAAGGGAAGAA; attB1REV_NCR084, GGGGACTGCTTTTTTGTACAAACTTGTATTTTTCTCCCTTTACATG; attB4FWD_NCR121, GGGGACAACTTTGTATAGAAAAGTTGTCCTTCTATGCATGTTCAAA; attB1REV_NCR121, GGGGACTGCTTTTTTGTACAAACTTGGTTTTTCCCTCTTTATAGGT.

Entry clones for the GUS ORF and the 35S terminator were obtained in the Gateway vectors pDONR221 and pDONRP2R-P3, respectively [8]. Entry clones were recombined in the binary vector pKm43GW [66]. Leaf explants from the *M. truncatula* line R108 were transformed using *Agrobacterium tumefaciens* according to the method described in Cosson et al. [67].

For GUS analysis, three independent T2 transgenic lines were each time analyzed to avoid positional effects of the transgene insertion. No pattern variations were observed between independent lines. Untransformed plants and the constitutive GUS line pG3.3 (35S promoter fused to GUS) [68] were used as negative and positive controls respectively. For nodulation kinetics, R108 plants were cultivated on BNM agar plates and inoculated with OD_600_ = 0.1 suspensions of *S. meliloti* strain 1021 or *Sinorhizobium arboris* strain B554 (synonymous strain names LMG 14919 and HAMBI 1552) [69] which is an excellent symbiont of *M. truncatula* R108 forming numerous large, nitrogen fixing nodules. Samples were collected at indicated time points and embedded in 6% agarose. Tissue sections of 70 μm were prepared with a Leica VT1200S vibratome. GUS staining was done as described [70] and was allowed to proceed for 1 h (Additional file 2: Figure S6). Overnight staining did not alter the expression patterns (data not shown). The pattern of expression of the NCR genes in nodules induced by both *Sinorhizobium* strains were very similar.

For all pathogen assays, plants were cultivated on perlite/sand (3/1 vol/vol) substrate and watered with a commercial nutrient solution. Six weeks old plants were transferred to a growth chamber with saturating humidity the day before the inoculations and remained in these conditions all along the assay. *Dickeya dadantii* 3937, *Pseudomonas syringae* pv. *tomato* DC3000 and its *hrcC* derivative strain were cultivated at 30°C in LB medium. Inocula of OD_600_ = 0.1 were resuspended in 10 mM MgCl_2_ and were syringe infiltrated in the terminal leaflet of 5–8 leaves per plant. Sterile 10 mM MgCl_2_ solution was infiltrated as mock control. *Botrytis cinerea* strain B05.10 was cultivated on PDA medium [71] at 20°C. Spores were collected in ½ potato dextrose broth with 0.01% Tween 20 and inocula were normalized to 10^6^ spores/mL using a Malassez cell. Five microliter drops of mock/inoculum were put on 5 to 8 terminal leaflets per plant. Symptoms were scored at 1, 2 or 7 dpi and leaflets were collected for GUS staining. For wounding experiments, the terminal leaflet of 5–8 leaves per plant were pinched with forceps and collected 24 hours post wounding. Staining for all infections or treatments was allowed for 24 hours in the GUS staining solution at 37°C. The leaflets were transferred to bleach to remove chlorophyll before photographing.

### Antibodies and immunolocalization

The mature region of the NCR122 peptide was amplified from cDNA and cloned into the expression vector pBADgIII/A (Invitrogen). Recombinant proteins were purified according to the manufacturer’s instructions and used for immunization of rabbits by a commercial service (Agro-bio). Immunolocalisations were done exactly as described before [8]. For the SYTO13 nucleic acid staining, nodules sections were incubated for 5 minutes with 1 μM SYTO13 in H_2_O. Immuno- or SYTO13-stained sections were mounted in deionised water for confocal imaging. Fluorescence images were acquired at 1024×1024 pixels resolution with the confocal laser scanning microscope TCS SP2 from Leica, using 10X water-immersion and 63X oil-immersion objectives and Leica software. Images were processed with Adobe Photoshop for adjustment of contrast and brightness.

## Electronic supplementary material

Additional file 1: Table S1: NCR probe-sets and expression data. The NCR probe-set annotations are from the Affymetrix Medicago GeneChip. The expression data were extracted from MtGEA, NCBI GEO, and from the spatially-resolved nodule dataset. (XLSX 4 MB)

Additional file 2: Figure S1: Heat map of *S. meliloti* gene expression in the MtGEA compendium. **Figure S2.** Expression profile examples of 7 NCR genes. **Figure S3.** NCR gene expression in response to Nod factors and Myc factors. **Figure S4.** Representative clusters of spatial and temporal NCR expression profiles. **Figure S5.** Effect of Phosphinothricin treatment on NCR gene expression. **Figure S6.** Promoter-GUS analysis of NCR genes in nodules. **Figure S7.** Immunolocalization of NCR122 in nodule sections. **Figure S8.** NCR expression in plant tissues. **Figure S9.** NCR expression in response to phytohormones. **Figure S10.** Expression of NCR genes during microbial infections and elicitor treatment. **Figure S11.** Expression of NCR genes during drought and salt stress. **Figure S12.** Promoter-GUS analysis of NCR genes in infected and wounded leaves. **Figure S13.** Comparison of the spatial and temporal NCR expression profiles. (DOCX 5 MB)

Additional file 3: Table S2: Entropy of gene expression. The 9,000 probe-sets with the lowest entropy. (XLSX 3 MB)
