# Supplementary material for: Extreme specificity of NCR gene expression in Medicago truncatula
Source: BMC Genomics. 2014 Aug 25;15(1):712. doi: 10.1186/1471-2164-15-712 (PMC4168050; doi:10.1186/1471-2164-15-712)
Supplement: Supplementary file 2 — Additional file 2: Figure S1: Heat map of S. meliloti gene expression in the MtGEA compendium. Figure S2. Expression profile examples of 7 NCR genes. Figure S3. NCR gene expression in response to Nod factors and Myc factors. Figure S4. Representative clusters of spatial and temporal NCR expression profiles. Figure S5. Effect of Phosphinothricin treatment on NCR gene expression. Figure S6. Promoter-GUS analysis of NCR genes in nodules. Figure S7. Immunolocalization of NCR122 in nodule sections. Figure S8. NCR expression in plant tissues. Figure S9. NCR expression in response to phytohormones. Figure S10. Expression of NCR genes during microbial infections and elicitor treatment. Figure S11. Expression of NCR genes during drought and salt stress. Figure S12. Promoter-GUS analysis of NCR genes in infected and wounded leaves. Figure S13. Comparison of the spatial and temporal NCR expression profiles. (DOCX 5 MB) [file 12864_2014_6439_MOESM2_ESM.docx]

**BMC Genomics**

**Extreme specificity of NCR gene expression in *Medicago truncatula***

Ibtissem Guefrachi, Marianna Nagymihaly, Catalina I. Pislariu, Willem Van de Velde, Pascal Ratet, Mohamed Mars, Michael K. Udvardi, Eva Kondorosi, Peter Mergaert and Benoît Alunni

**Supplemental information; Additional file 2, Figures S1 to S13**

**
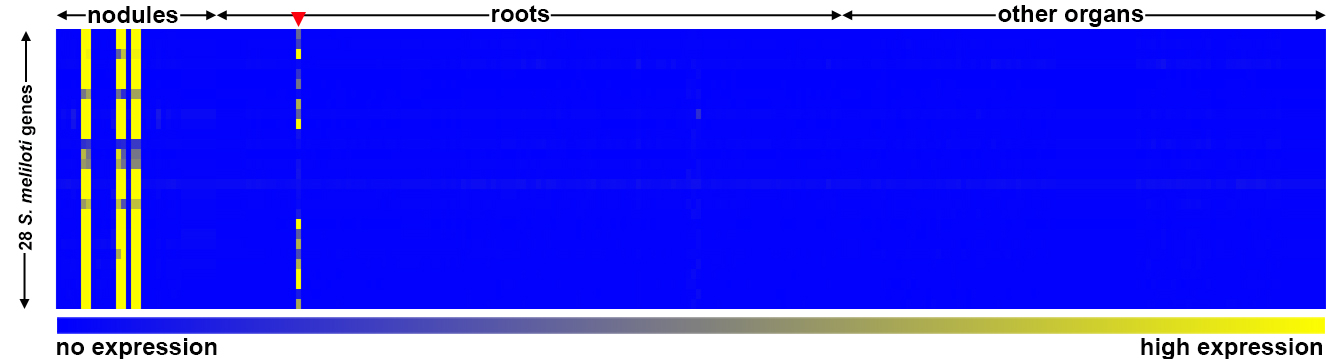
Figure S1 Heat map of *S. meliloti* gene expression in the MtGEA compendium.** The Affymetrix gene Chip contains probe-sets for monitoring the expression of the *S. meliloti* genes. The *S. meliloti* probe-set can thus reveal whether samples of the MtGEA contain this bacterium. The heat map shows the expression of 28 *S. meliloti* genes (rows) in 267 experimental conditions (columns). The probe-set included 28 genes of the locus carrying the *nifHDK* operon encoding the nitrogenase enzyme and the dataset used for the generation of the heat map is provided in Additional file 1: Table S1. Experiments are ordered as in Figure 1 and as indicated above the columns. The color scale bar indicates the expression level from background level (blue) to maximum level (yellow).

The red arrowhead indicates a mycorrhizal sample that is contaminated with nodules. The expression of the NCR gene set in mycorrhizal roots was inconsistent with our previous results [14] and also with other mycorrhizal samples present in the MtGEA compendium in which the NCR genes are not expressed. Analysing the 28 *S. meliloti* probe-sets, we found that these genes were expressed in nodule samples, as expected, as well as in the particular mycorrhizal sample in which the NCRs are also active, while in all other samples including other mycorrhizal samples, roots and other organs, the *S. meliloti* probe-sets gave background signals. Considering that the nitrogenase locus is known to be exclusively expressed in nodule bacteroids [Becker et al., *Mol Plant –Microbe Interact* 2004, **17**:292-303], this mycorrhizal sample is most likely impure and contaminated with nodules.

**
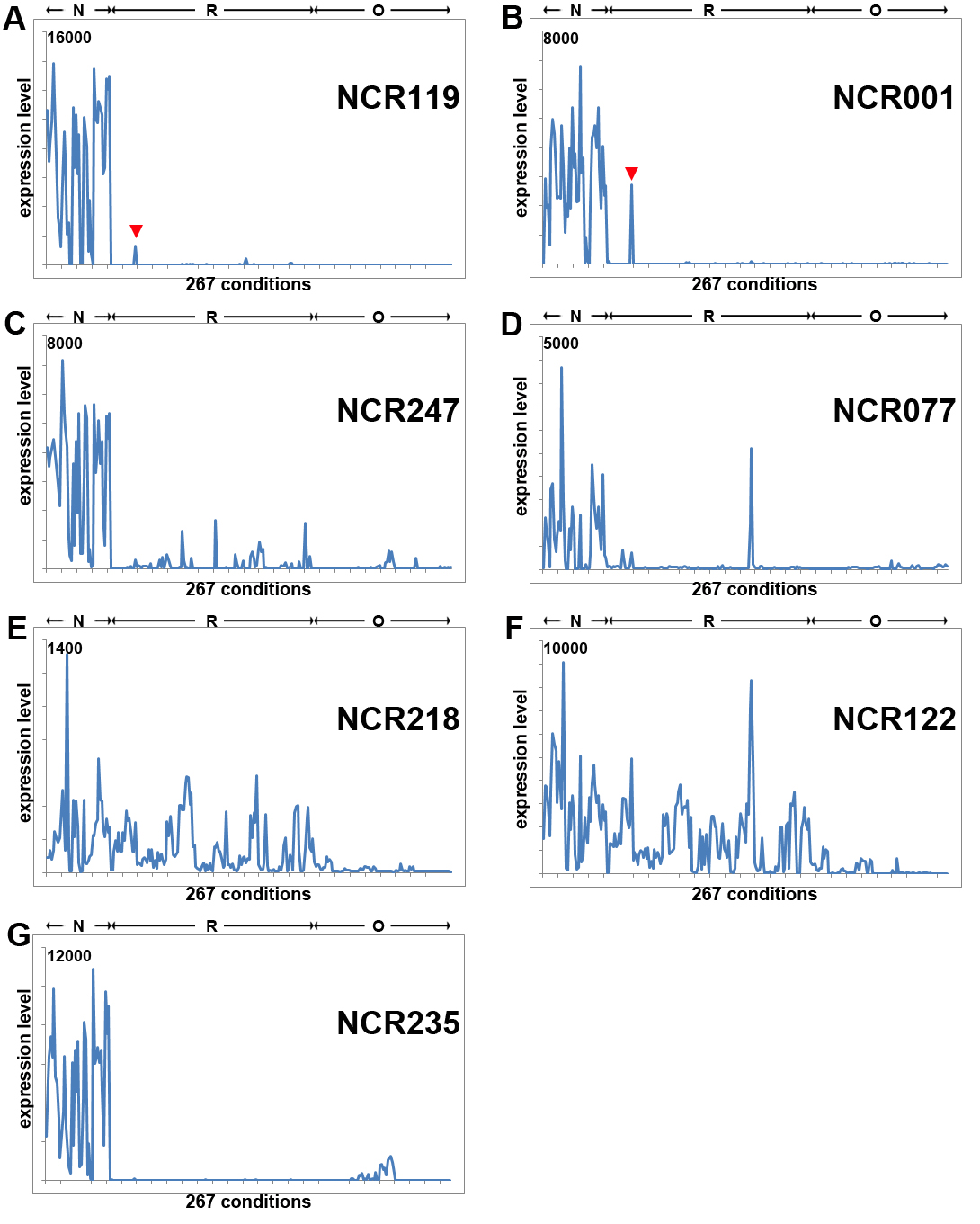
Figure S2 Expression profile examples of 7 NCR genes. (A-G)** The expression pattern of 7 individual NCR genes in 267 experiments (x-axis) is provided. The experiments are ordered as in Figure 1. N is nodules; R is root conditions; O is all other conditions. The y-axis is the expression level according to the fluorescence hybridization signal and is scaled according to the maximum hybridization signal as indicated on the top of the axis. The red arrowheads in panels A and B indicate the mycorrhizal sample that is contaminated with nodules.

**
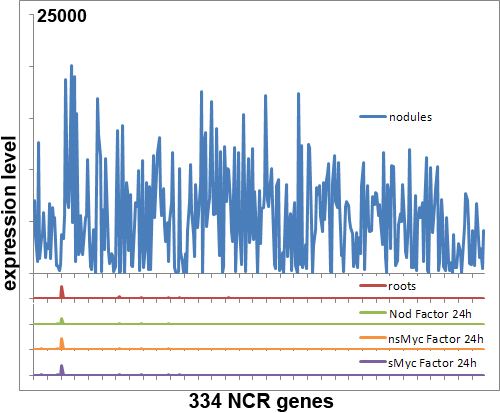
Figure S3 NCR gene expression in response to Nod factors and Myc factors.** The expression pattern of 334 NCR probe-sets (x-axis) is shown for nodules 10 dpi, untreated control roots, 24h 10^-8^M Nod factor treatment, 24h 10^-7^M non-sulfated Myc factor treatment (nsMyc) and 24h 10^-8^M sulfated Myc factor treatment (sMyc) [25]. The y-axis represents the strength of the hybridization signal and the graphs for all treatments are at the same scale (maximum 25,000).

**
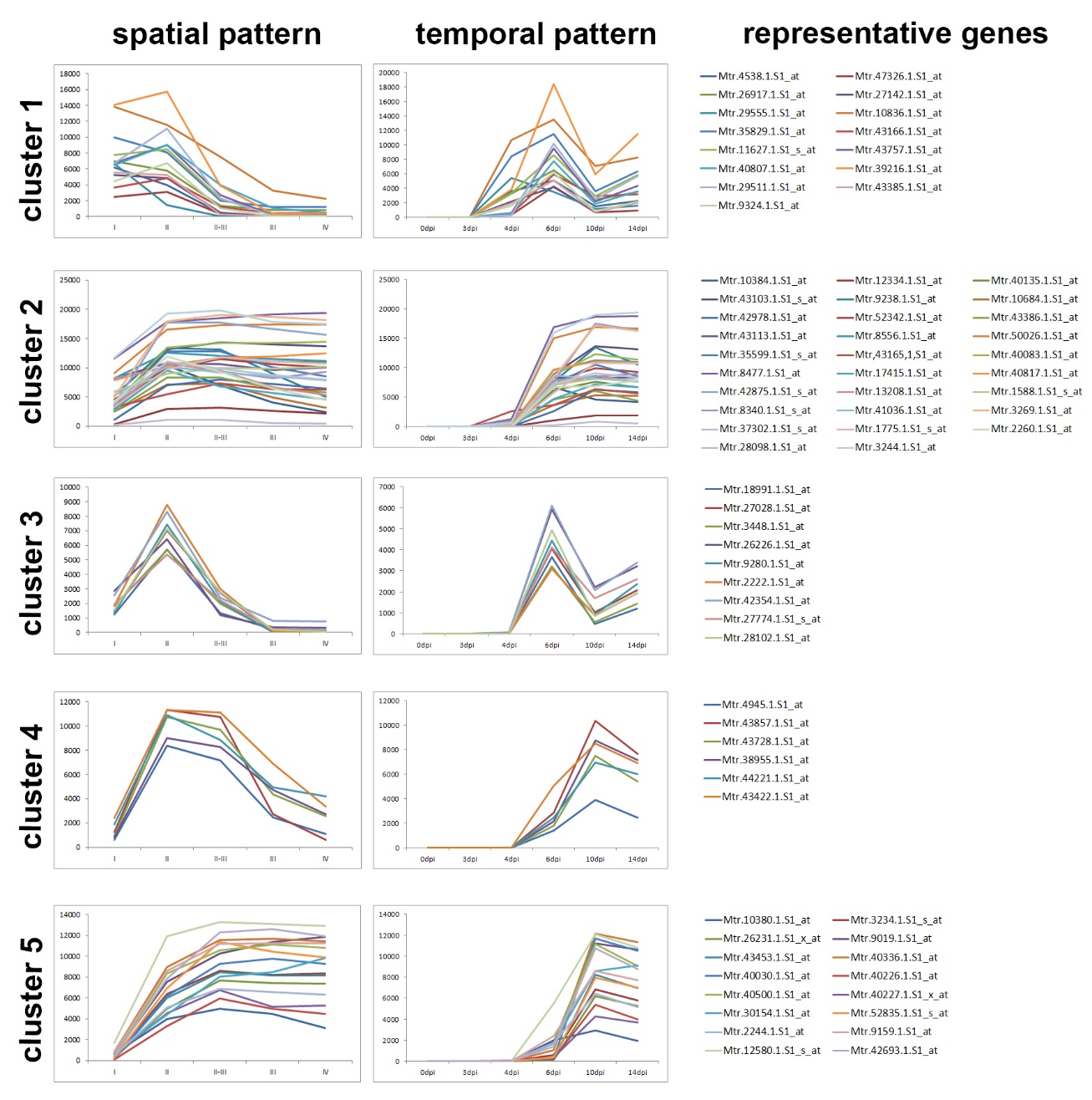
Figure S4 Representative clusters of spatial and temporal NCR expression profiles.** Five representative expression profiles are shown (cluster 1 to 5). The spatial pattern (left), the temporal pattern (centre) and the genes constituting the cluster (right) are shown. Genes in cluster 1 display the highest expression in the nodule apex sample I, are still high in sample II and then decline rapidly (Additional file 3: Figure S5). Cluster 2 genes are already activated in sample I but their expression increases in sample II and remains high in the whole nodule. Cluster 3 genes have a very sharp pattern and are highly expressed in sample II, low in sample I and II-III and close to zero in the samples III and IV. The genes of cluster 4 have a similar profile but are expressed in a broader zone with high levels of expression in samples II and II-III. Finally, cluster 5 genes are absent in sample I, partially activated in sample II and then fully activated in sample II-III, III and IV. The genes are identified by their probe-set annotation number as provided in Additional file 1: Table S1.

**
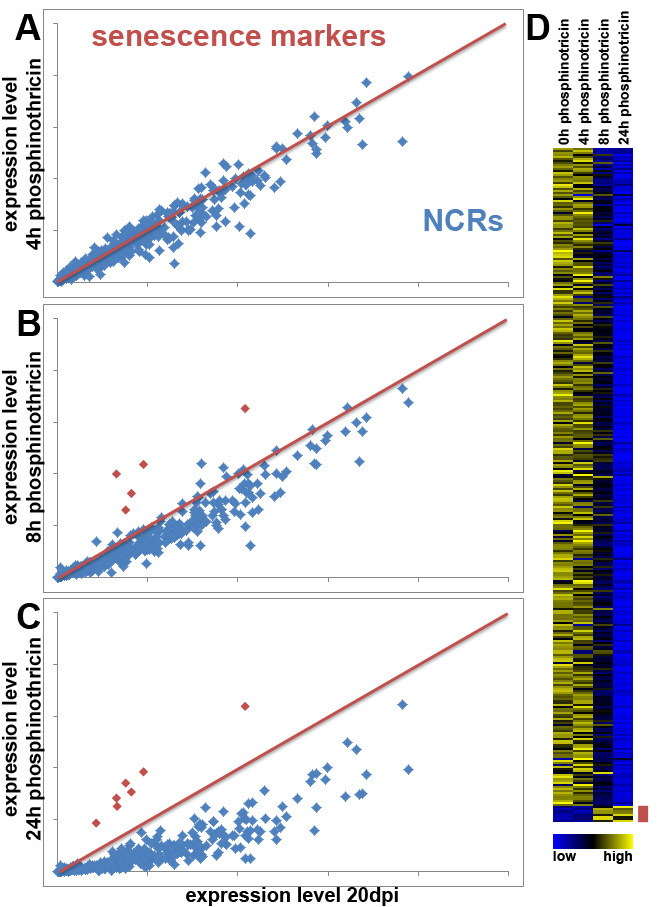
Figure S5 Effect of Phosphinothricin treatment on NCR gene expression. (A-C)** Scatter plot of gene expression levels in 20 dpi nodules compared to 20 dpi nodules 4h **(A)**, 8h **(B)** and 24 h **(C)** post treatment with the herbicide phosphinothricin [29]. A set of senescence markers from Van de Velde et al. [28] (red dots) and the 334 NCR genes (blue dots) show opposite regulation from 8h post treatment. The red line in the three graphs indicates a ratio of 1 between the two conditions. The probe-sets for the senescence marker genes, encoding cysteine proteinases, a chitinase, a nuclease a nucleoside transporter and a metal-nicotinamide transporter, are provided in Additional file 1: Table S1. **(D)** Heat map of NCR and senescence markers expression before and 4, 8 and 24 h post phosphinothricin treatment. Expression levels vary from low (blue) to high (yellow). The red bar next to the heat map indicates the location of the senescence marker genes.

**
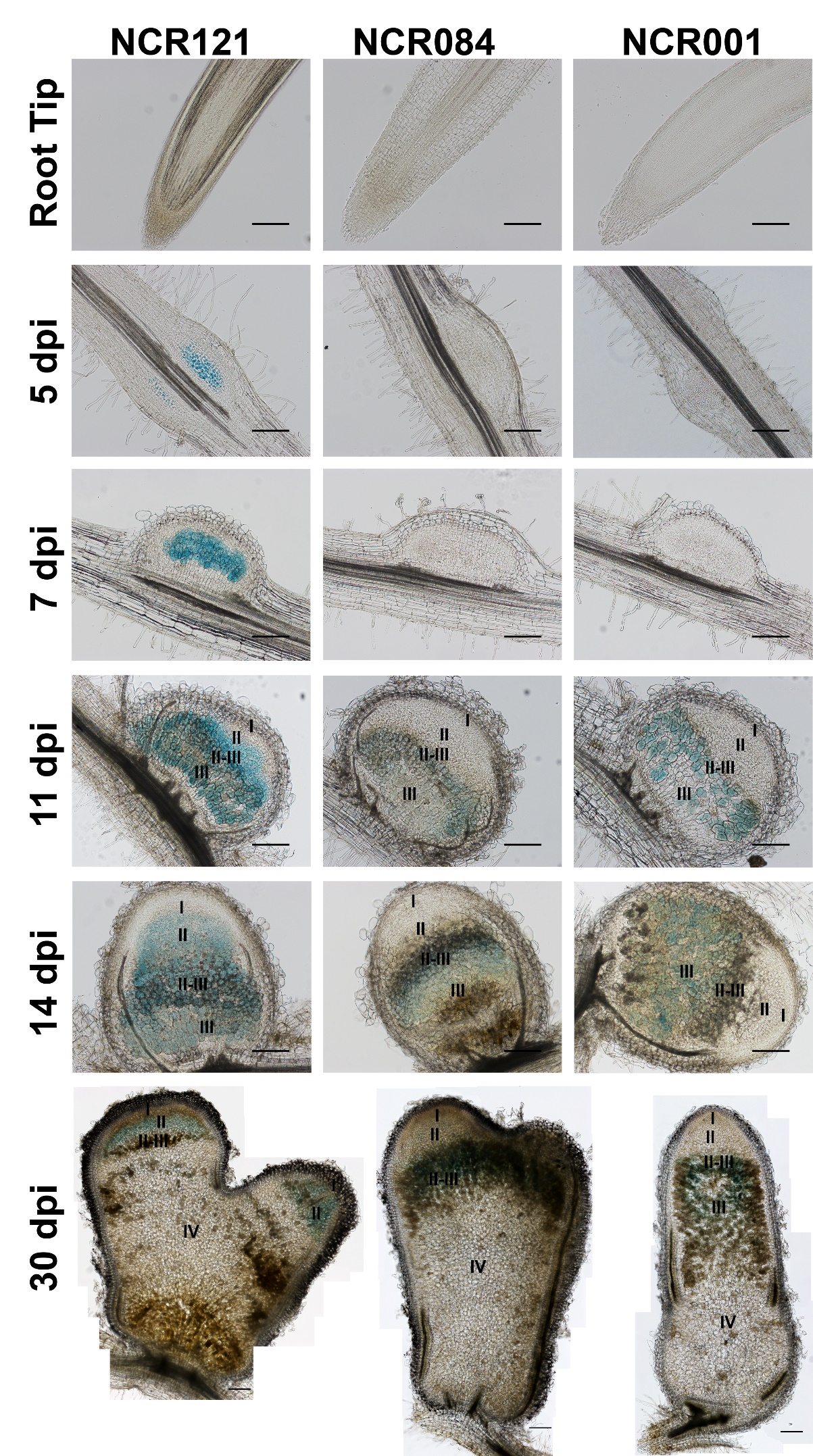
**

**Figure S6 (previous page) Promoter-GUS analysis of NCR genes in nodules.** Sections (70 µm) of root tips and nodules of different ages of transgenic plants carrying promoter-GUS fusions for the NCR121, NCR084 and NCR001 genes were stained for GUS activity observed as blue color. Scale bars are 50 µm. The location of the nodule zones in mature nodules is indicated: I is the meristem; II is the infection zone; II-III is the interzone; III is the fixation zone and IV is the senescence zone. The nodules shown in the figure were induced by *S. arboris* strain B554 but indistinguishable patterns were obtained in nodules formed by *S. meliloti* strain 1021 (data not shown).

**
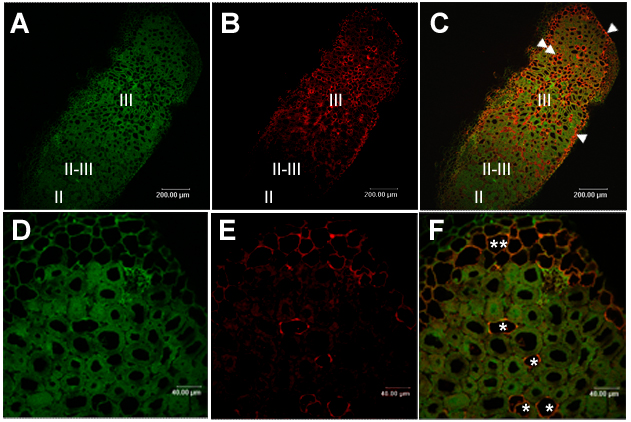
Figure S7 Immunolocalization of NCR122 in nodule sections.** Sections of nodules were stained with the DNA label SYTO13 (green) to reveal the bacteria **(A,D)** and were immunolabelled with the anti-NCR122 antibody (red) **(B,E)**. **(C,F)** Overlays of **(A,B)** and **(D,E)** respectively. **(D-F)** Enlargement of the top part of the nodule shown in **(A-C)**. Arrowheads or double asterisks mark NCR122-labeled cells in the nodule cortex and the double arrowhead or asterisks mark uninfected cells of the nitrogen fixation zone III labeled by NCR122. II is the infection zone; II-III is the interzone. Scale bars are 200 µm in panels A-C and 40 µm in panels D-F.

**
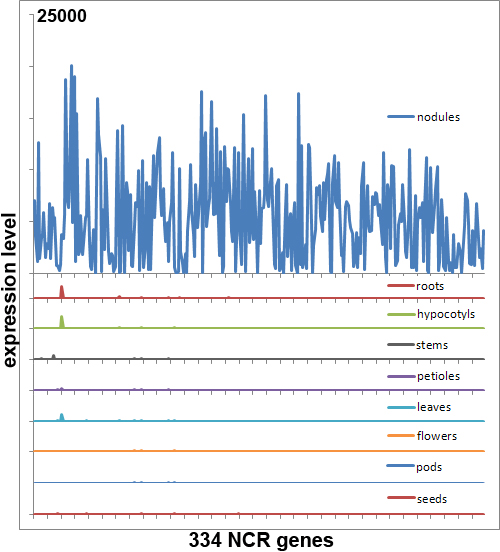
Figure S8 NCR expression in plant tissues.** The expression pattern of 334 NCR probe-sets is shown for nodules, roots, hypocotyls, stems, petioles, leaves, flowers, seed pods and seeds [20]. The graphs for all organs are at the same scale (maximum hybridization signal value is 25,000). The small peaks that can be seen in some of the organ samples correspond to the NCR genes with relaxed expression (Figure S2).

**
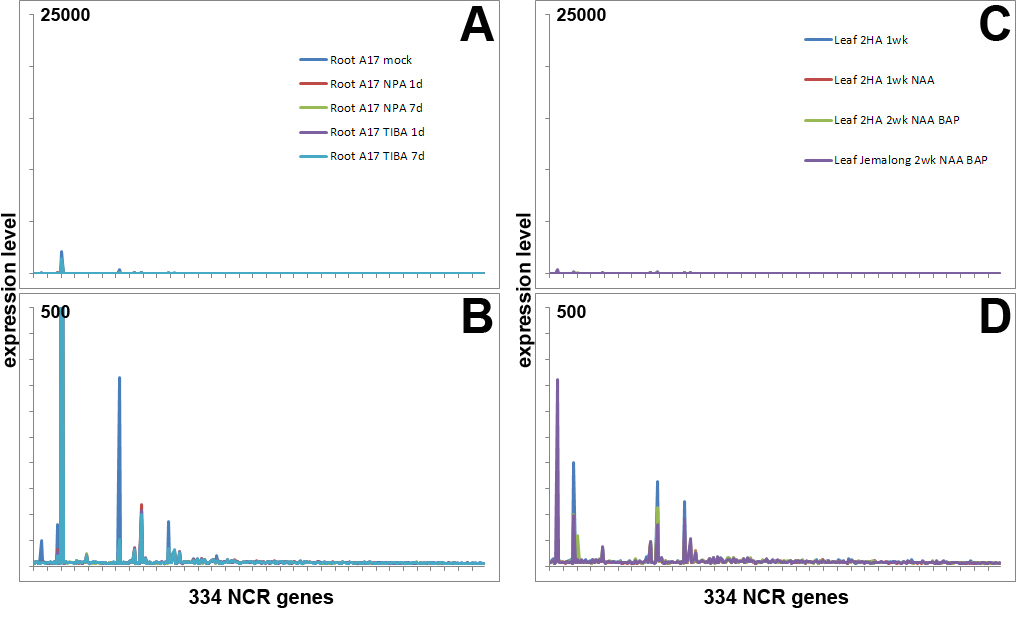
Figure S9 NCR expression in response to phytohormones. (A,B)** NCR expression after treatments for 1 (1d) or 7 days (7d) of roots with the auxin transport inhibitors naphthylphthalamic acid (NPA; 200 µM) and 2,3,5-triidobenzoid acid (TIBA; 200 µM) [33]. **(C,D)** NCR expression after treatments of leaves for 1 (1wk) or 2 (2wk) weeks with the auxin 1-naphthaleneacetic acid (NAA; 10 µM) or the cytokinin 6-benzylaminopurine (BAP; 4 µM) [32]. (A) and (B) on the one hand and (C) and (D) on the other hand represent the same data but the scale of the graphs are at relative expression level 25,000 maximum in (A) and (C) and at maximum level 500 in (B) and (D).

**
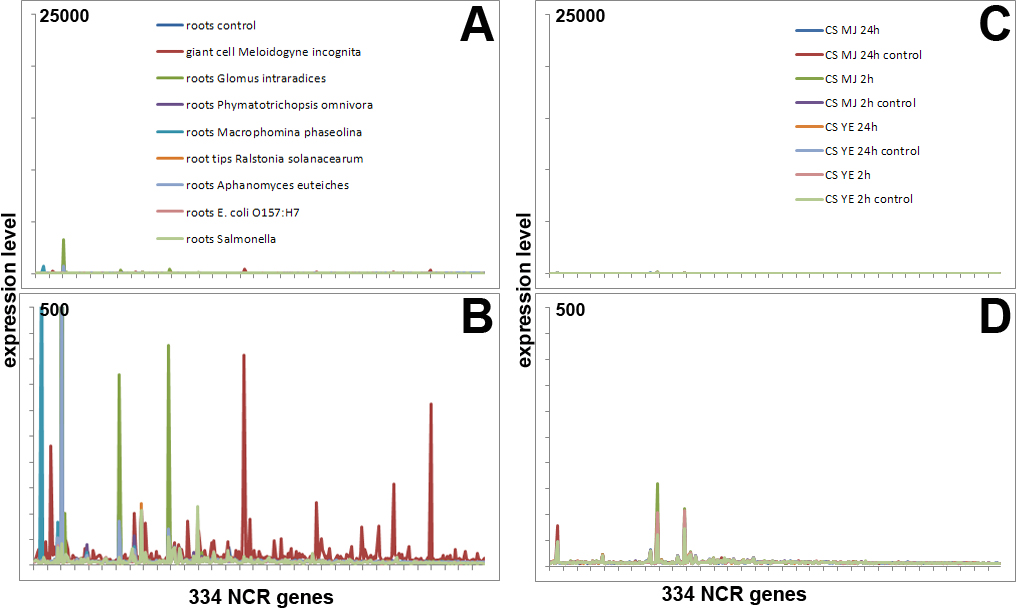
Figure S10 Expression of NCR genes during microbial infections and elicitor treatment. (A,B)** NCR expression in *M. truncatula* control roots and roots infected with the pathogenic fungi *Phymatotrichopsis omnivora* [38] and *Macrophomina phaseolina* [39] and the symbiotic mycorrhizal fungus *Glomus intraradices* [40], the oomycete *Aphanomyces euteiches* [41], the bacterial pathogen Ralstonia solanacearum, the nematode *Meloidogyne incognita* [42] and the human enteric bacterial pathogens *Escherichia coli* O157:H7 and *Salmonella enterica* which are frequent sources of legume food contamination and are capable of surface and internal colonization of *M. truncatula* roots [22]. **(C,D)** NCR expression in a *M. truncatula* cell suspension derived from root cells (CS) after treatments for 2h or 24h with the defense response-inducing signals methyl jasmonate (500 µM) (MJ) and yeast elicitor (50 µg glucose equivalents ml^-1^) (YE) [43]. (A) and (B) on the one hand and (C) and (D) on the other hand represent the same data but the scale of the graphs are at relative expression level 25,000 maximum in (A) and (C) and at maximum level 500 in (B) and (D).

**
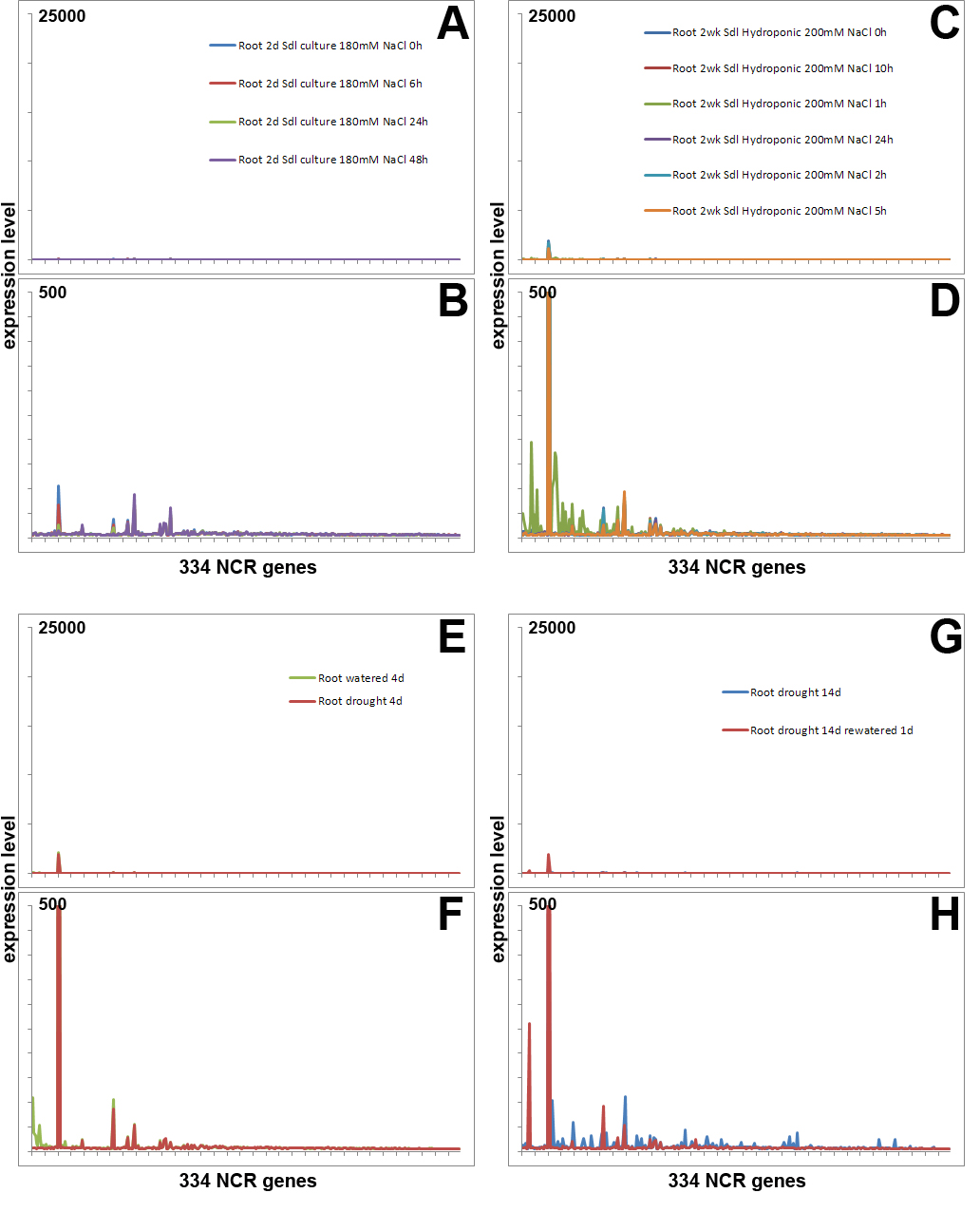
Figure S11 Expression of NCR genes during drought and salt stress. (A-H)** NCR expression in roots exposed to NaCl salt stress [44] or drought stress [45]. (A) and (B), (C) and (D), (E) and (F) and (G) and (H) represent pairwise the same data but the scale of the graphs is at relative expression level 25,000 maximum in (A), (C), (E) and (G) and at level 500 in (B), (D), (F) and (H).

**
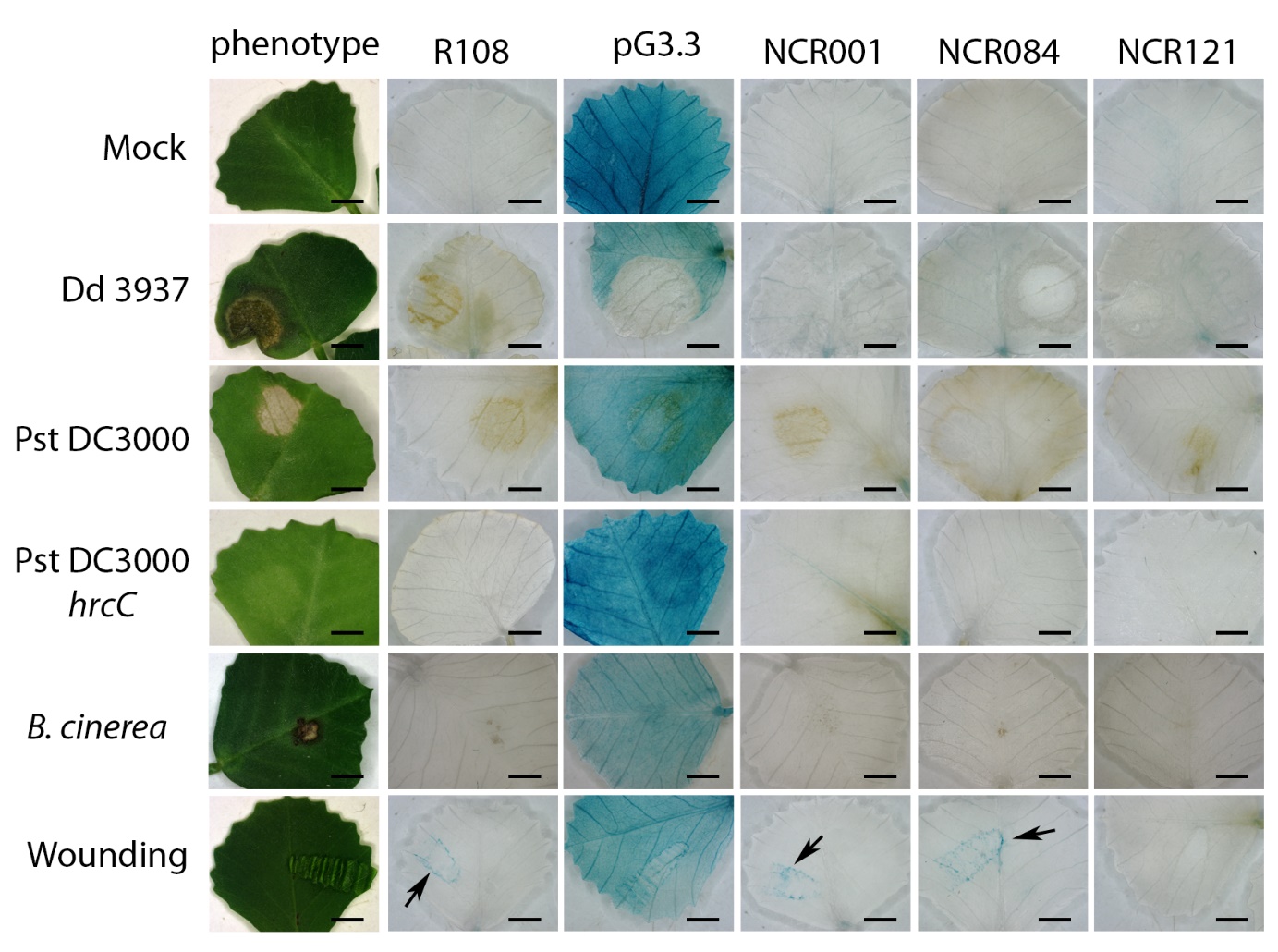
Figure S12 Promoter-GUS analysis of NCR genes in infected and wounded leaves.** Wild type *M. truncatula* R108 (R108) and transgenic R108 carrying NCR promoter-GUS fusions (NCR001, NCR084, NCR121) or a constitutive GUS under the control of the 35S promoter (pG3.3) were mock infected, infected with *Dickeya dadantii* 3937 (Dd 3937), *Pseudomonas syringae pv. tomato* DC3000 (Pst DC3000), the *Pseudomonas syringae pv. tomato* DC3000 *hrcC* TTSS mutant (Pst DC3000 *hrcC*) or *Botrytis cinerea* (*B. cinerea*), or wounded and then stained for GUS activity, which is observed as blue color. Note that in certain leaves, the leaf base and the veins are faintly stained blue. Also the wounding print is slightly stained (arrows). This staining is also observed in untransformed R108 and thus represents background signal. The left panels show the leaf phenotype after the treatment and before the staining procedure. Scale bars are 2 mm.

**
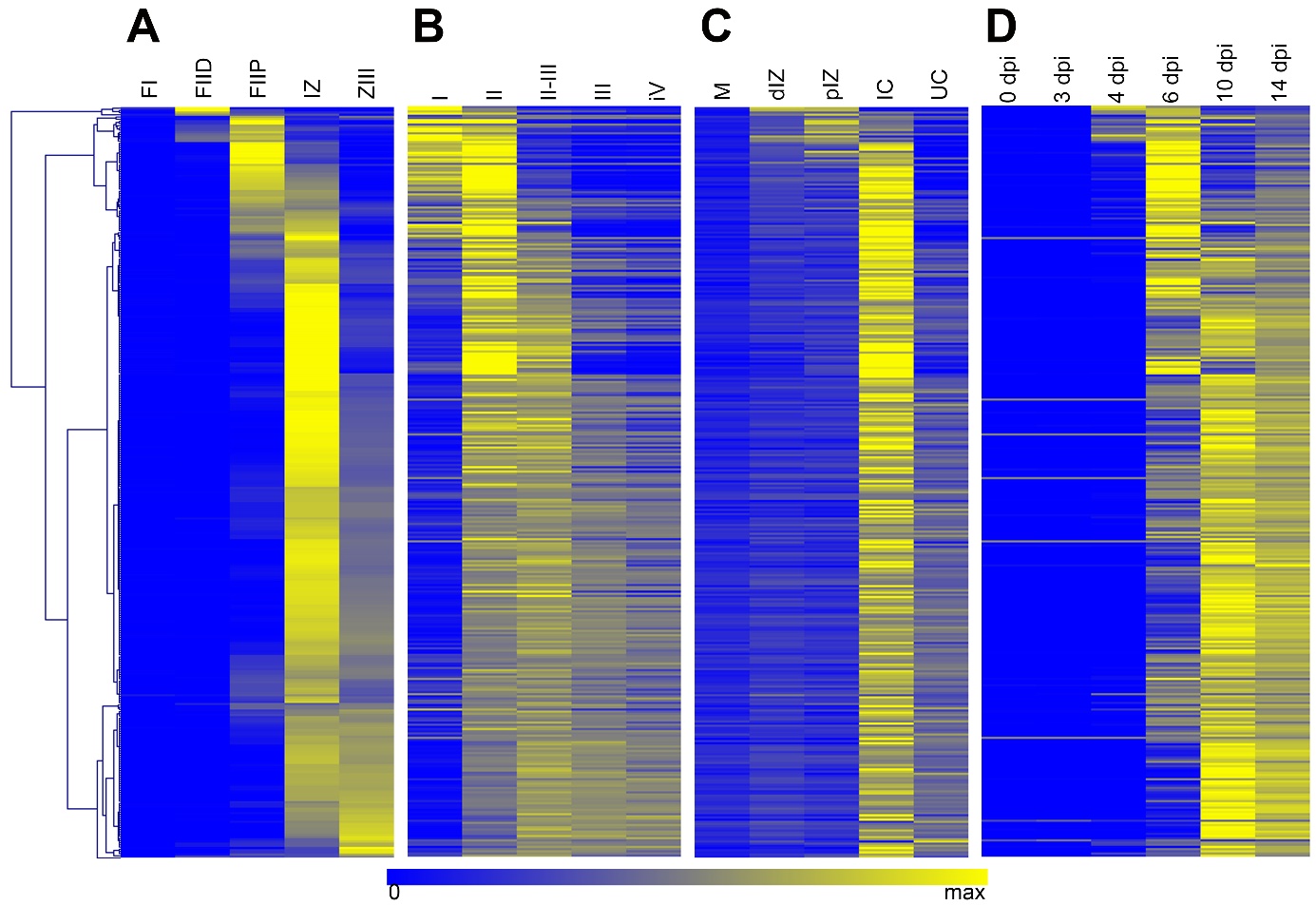
Figure S13 Comparison of the spatial and temporal NCR expression profiles. (A)** The expression profiles for the NCR gene set described in this study were extracted from the data obtained by laser-capture microdissection coupled to RNA-Seq from the website https://iant.toulouse.inra.fr/symbimics [54]. FI corresponds to meristem, FIID to the distal zone II or infection zone, FIIP to the proximal zone II, IZ to the interzone between the infection zone and the fixation zone and ZIII to the fixation zone. Clustering was performed using Pearson correlation. **(B)** The corresponding profiles obtained by hand-dissection of nodules in this study. Samples I, II, II-III, III and IV correspond to the nodule tissues from the most apical part of the nodule with the youngest symbiotic cells to the most proximal part containing the oldest symbiotic cells. **(C)** The corresponding profiles obtained by laser-capture microdissection coupled to Affymetrix microarray analysis described by Limpens et al. [23]. M corresponds to meristem, dIZ to the distal zone II or infection zone, pIZ to the proximal zone II, IC to infected cell and UC to uninfected cell. **(D)** The corresponding profiles in function of nodule age (dpi). For all data sets, the expression patterns in the different dissected nodule zones are expressed in percentage from their total. The NCR genes are ordered identically in all 4 panels according to a hierarchical clustering of the dataset from panel (A).
